# Panic disorder during pregnancy and the first three years after delivery: a systematic review

**DOI:** 10.1186/s12884-024-07127-1

**Published:** 2025-01-17

**Authors:** F. Gerrik Verhees, Antonia Bendau, Stefanie Unger, Katharina L. Donix, Eva Asselmann, Julia Martini

**Affiliations:** 1 Department of Psychiatry & Psychotherapy, Faculty of Medicine, Carl Gustav Carus University Hospital, Technische Universität Dresden, Dresden, Germany; 2 Faculty of Health, HMU Health and Medical University, Potsdam, Germany; 3 Faculty of Medicine, Institute and Policlinic of Occupational and Social Medicine, Carl Gustav Carus University Hospital, Technische Universität Dresden, Dresden, Germany; 4 Institute of Clinical Psychology and Psychotherapy, Technische Universität Dresden, Dresden, Germany

**Keywords:** Anxiety/panic disorder, Panic, Pregnancy, Postpartum, Peripartum, Maternal health, Dyadic

## Abstract

**Background:**

Panic disorder (PD) is highly prevalent during the peripartum period. The aim of this systematic review was to summarize evidence on risk factors and course patterns of peripartum PD as well as maternal, infant or dyadic outcomes during the first three years after delivery.

**Methods:**

A literature search was conducted according to PRISMA guidelines. Inclusion criteria were: (1) a diagnosis of PD or panic attacks during pregnancy, (2) risk factors and course as well as maternal, infant or dyadic outcomes measured in pregnancy and/or up to 3 years postpartum (3) peer-reviewed articles in English or German published between 1980 and April 2024. After screening of *n* = 2,740 records, *n* = 75 records based on *n* = 64 projects were eligible for this systematic review.

**Results:**

Overall, *n* = 47 studies investigated the course of PD during the peripartum period, *n* = 23 studies examined the associations of PD and obstetric, neonatal or infant outcomes, and *n* = 5 studies focused on the associations of PD and characteristics of the mother-infant dyad. We found (1) no common trajectory, but heterogeneous courses of maternal PD in the peripartum period, (2) associations of maternal PD with birth complications and subsequent postpartum depression, and (3) evidence for associations of PD with infant and dyadic outcomes.

**Limitations:**

Diverse outcome measures in recent original publications did not allow for a meta-analytic approach.

**Conclusion:**

Heterogenous courses and outcomes of peripartum PD require comprehensive monitoring of affected mothers and their infants. There is a need for further longitudinal investigations into familial transmission of anxiety disorders.

**Supplementary Information:**

The online version contains supplementary material available at 10.1186/s12884-024-07127-1.

## Introduction

Panic disorder (PD) is one of the most prevalent and disabling psychiatric disorders [7, 51, 131] that emerges prior to or during the reproductive years with a mean age of onset around 30 years and a 12-months prevalence of 2.8% in women [51, 61]. Investigating its complex phenomenology as well as peripartum outcomes is crucial to understand the effects of PD on (expectant) mothers and their infants.

Recently, two meta-analyses on the *prevalence* of anxiety disorders including PD in the peripartum period were published. Results indicated heterogeneous courses of PD (Table 1). Viswasam and colleagues found no significant difference in the pooled prevalence of PD between the first (3%, 95%CI: 2–5%) and the third (3%, 95%CI: 2–6%) trimester of pregnancy in more than 5,000 pooled participants [122]. In the postpartum period, a slightly lower pooled prevalence rate of 1.7% (95%CI: 0.1–2.8%) was reported in more than 14,000 participants by Goodman et al. [40]. Another meta-analysis examined the prevalence of perinatal anxiety disorders in low- and middle-income countries and revealed comparable prevalence rates for PD during the peripartum period (3.7%, 95%CI: 2.1–5.6) [96].

**Table 1:** Epidemiology of panic disorder in pregnancy and postpartum

	Viswasam et al. [122]	Goodman et al. [40]	Roddy Mitchell et al. [96]
**Systematic Search**	Google Scholar, MEDLINE, EMBASE, PsychInfo, PubMed, ProQuest (till 2019)	MEDLINE, PsychInfo, PubMed, CINAHL (till 2013)	Embase, MEDLINE, PsycINFO, Cochrane Library, CINAHL, and Web of Science (till 2023)
	**Prevalence in %**	**Prevalence in %**	**Prevalence in %**
	**Pregnancy**	**Postpartum**	**Peripartum**
**PD**,** prevalence range (number of studies)**	0.4–7.5 (12)	0.5–3.4 (6)	0.4–15.3 (13)
**PD**,** pooled prevalence (95%CI**,** I**^**2**^**)**	3 (2–4, I^2^ = 79%)	1.7 (0.1–2.8, I^2^ = 89%)	3.7 (2.1–5.6, I^2^ = 91%)

A substantial number of studies investigated trajectories of peripartum PD. However, different methods, inclusion criteria and assessment periods were applied. Some early prospective-observational studies found decreasing or low rates of PD during pregnancy [8, 38, 49, 56, 84, 121] and described this course as the “classical presentation”. At the same time, unchanged or even worsening course of PD during pregnancy has also been reported [18, 20, 42, 130]. Other evidence suggested heterogeneous trajectories during the postpartum period (e.g. exacerbation of PD) and an elevated risk for new onset PD [8, 18, 20, 49, 102]. It has also been hypothesized that breastfeeding may be relevant for the course of PD, although studies have been inconclusive [8]. More recently, PD has also been discussed as a prominent risk factor for postpartum depression in early motherhood [69, 72, 105].

In addition to its clinical presentation during the peripartum period, it is of particular interest what maternal and infant outcomes result from PD during pregnancy and the first couple of years after delivery. Recent evidence shows that perinatal anxiety disorders are associated with adverse obstetric, neonatal, and infant outcomes [4, 65, 79, 90, 108], but studies often do not focus on PD in detail. However, the role of PD might be particularly important, since it was hypothesized that panic attacks during pregnancy may lead to fetal exposure to stress hormones, and some studies actually suggest that PD is associated with pregnancy-related complications and adverse infant outcomes [2, 9, 14, 17, 107, 125, 134]. Recent research has shown conflicting results regarding the association between peripartum PD and adverse birth outcomes such as preterm birth, low birth weight, and mode of delivery [9, 14, 134]. Furthermore, early signs of infant anxiety (such as behavioral inhibition) that are supposed to be associated with later anxiety disorders have been studied mainly in infants aged 2 years and older [24, 32, 99]. Research on further mother-infant dyadic outcomes such as mother-infant interaction, bonding/attachment, and parenting behavior is quite complex and diverse thus far.

Taken together, our recent knowledge on risk factors, incidence and course of PD during pregnancy and postpartum as well as maternal, obstetric, neonatal, infant and dyadic outcomes is complex and partially fragmentary [70]. Therefore, the aim of this systematic review was to provide a comprehensive overview of the literature on the course of peripartum PD and its outcomes to improve early detection and treatment.

### Research questions

What is the current evidence on maternal and obstetric (e.g. course of peripartum PD, gestational complications, maternal health, psychosocial outcomes), neonatal and infant (e.g. birth weight, gestational age, infant health) as well as mother-infant dyadic outcomes (e.g. duration of breastfeeding, quality of interaction, bonding/attachment, parenting behavior) of peripartum PD?

## Materials and methods

**Fig. 1:**
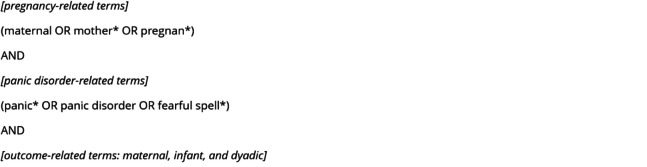
Complete search string adapted individually for PubMed, EBSCOhost, and Web of Science

A computerized literature search (Fig. 1) was conducted according to PRISMA using PubMed, EBSCOhost, and Web of Science. Inclusion criteria were: (1) a diagnosis of PD (by ICD, DSM or research diagnostic criteria) or panic attacks (without meeting full diagnostic criteria of PD) during pregnancy (as independent or dependent variable), (2) maternal, obstetric, neonatal, infant or dyadic outcome with outcomes measured in pregnancy, up to 3 years postpartum, or both, (3) peer-reviewed articles in English or German published from 1980 until April 2024, (4) case reports and series, cross-sectional, case control, prospective studies, and reviews. Additionally, articles cited in the identified papers were retrieved manually. Duplicates were excluded as well as articles that were not related to PD and pregnancy, bundled PD inextricably with other anxiety disorders, or reported results multiple times in different publications.

The review was performed based on the updated Preferred Reporting Items for Systematic Reviews and Meta-Analyses (PRISMA) statement [87]. All methods of search strategy, study selection, data extraction, quality estimation, and analysis were specified in advance and documented in a protocol [10].

**Fig. 2:**
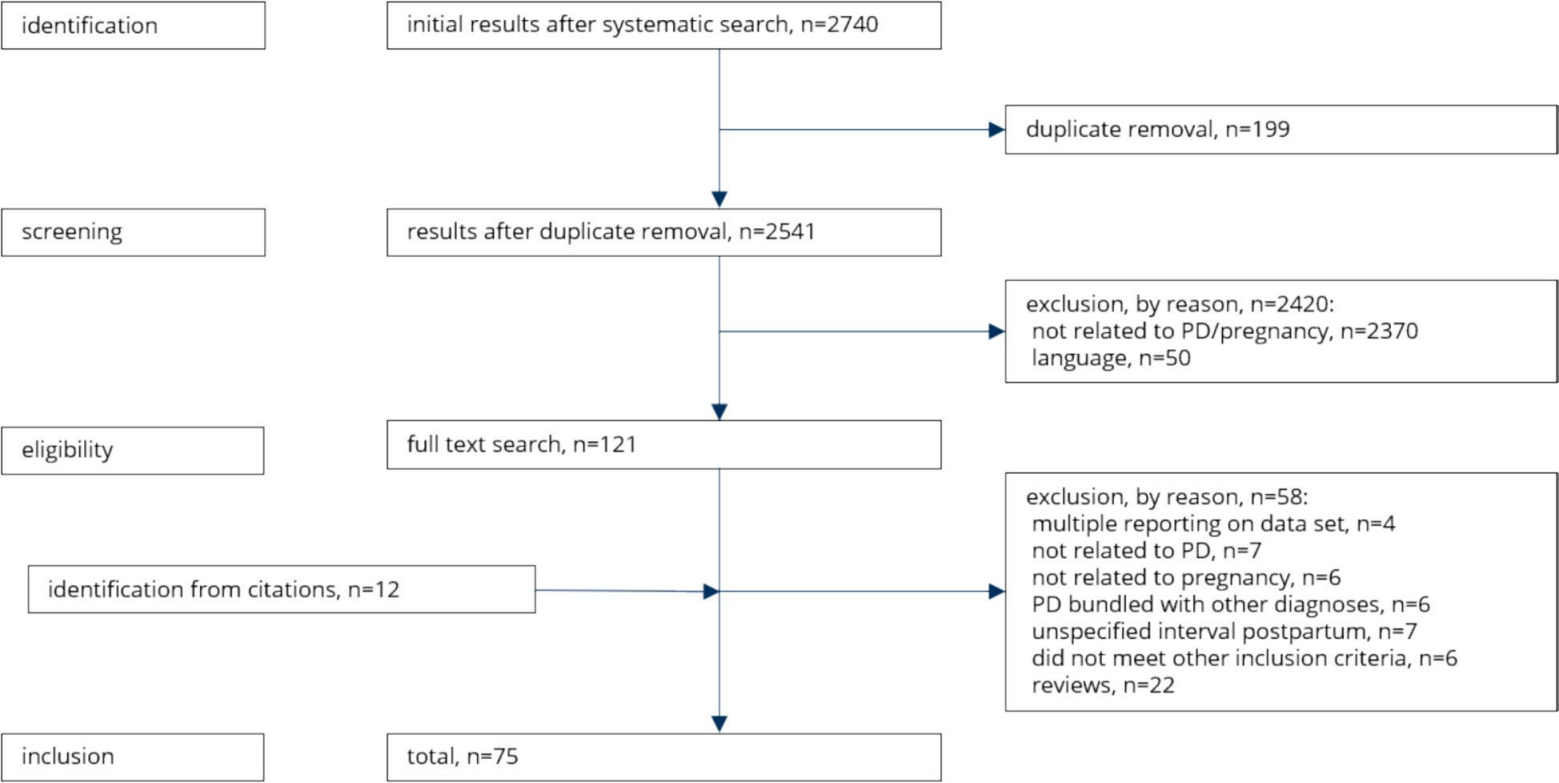
Detailed study flow chart, with exclusions by reason

Altogether, *n* = 2,740 records could be identified from electronic bibliographic sources (Fig. 2). After eliminating duplicates automatically and manually (*n* = 199), the remaining records were stepwise screened for inclusion and exclusion criteria. After extensive title and abstract screening, *n* = 2,420 records were excluded because they did not relate to PD and/or pregnancy (*n* = 2,370), or they were not written in English or German (*n* = 50).

Criteria decisions were made sequentially until reaching a possible point of exclusion. When any criterion could not be clearly determined, the full-text was consulted before categorizing. An additional set of *n* = 12 studies was identified from screened references. All publications could be retrieved and were available in full text. A detailed screening was performed by one of two independent reviewers (FGV, JM) for those records (*n* = 133).

Given that original publications on prevalence of peripartum PD were already comprehensively summarized in three systematic reviews/meta-analyses [40, 51, 96, 122, 131] we decided to only list and discuss original publications that were not included in these meta-analyses to update their findings (exclusions see Supplemental Table 1). Recent narrative reviews on perinatal anxiety disorders were thoroughly screened to ensure that all original papers that were included in these reviews were also identified by our search (list of respective reviews on request). We decided to take the original papers as the basis for this systematic review.

In total, *n* = 58 records were excluded, as detailed in Fig. 2. Thus, *n* = 75 records were eligible for qualitative synthesis involving *n* = 64 different projects.

### Data extraction and risk of bias estimation

A data extraction template was developed, pilot-tested in a randomized subsample of 10 articles and revised accordingly. Based on the revised template, information on study design and main results were extracted, as specified beforehand [10].

Abstract screening was performed by one out of four (AB, FGV, JM, SU), full text search by one out of two independent reviewers (FGV, JM) with uncertainties resolved by consensus between the reviewers. Independent screening and extraction by two different reviewers (FGV, SU) were carried out for a randomly selected sub-sample of 14 studies. An initial interrater agreement rate of 86% and concordance measured by Cohen’s κ of 0.84 was achieved, any remaining uncertainties were again resolved unanimously.

A subset of potential biases was specifically addressed via inclusion criteria. Recall bias was accounted for by only including studies with assessments shortly before, during, and/or after pregnancy (up to 3 years postpartum) or full clinical records. Publication bias was addressed by search of grey literature through Google Scholar with a reduced search string (“(pregnancy OR postpartum) AND (Panic OR Panic disorder): pdf”, April 17, 2024) and limited to the first 50 results, relying on the embedded relevance algorithm [39] and yielded no additional publications meeting our inclusion criteria. As no quantitative synthesis in form of a meta-analysis could be undertaken due to the variety and heterogeneity of outcome measures, no further appraisal of bias risks has been carried out. Methodological strengths and weaknesses of the original works are discussed below, where relevant.

## Results

A total of *n*= 75 studies representing 8 case reports and series, 24 cross-sectional, 13 case-control, 26 prospective longitudinal, and 4 retrospective studies were included in our analysis. Overall, of the cross-sectional studies 46% were conducted during pregnancy, 42% had assessments in the postpartum period and 12% recruited in either period (without specifying whether a history of PD prior to the current pregnancy was recorded). Only 2 longitudinal studies differentiated between new onset or recurrence of PD during pregnancy or postpartum [27, 74]. Most studies were conducted in Western countries (58 studies, 77%): Europe*n* = 23 studies with 65,418 participants (France: one study, *n* = 598; Germany: 4 studies, *n* = 433; Hungary: 4 studies, *n* = 60,944; Ireland: one study, *n* = 1; Italy: 8 studies, *n* = 2,582; Netherlands: two studies, *n* = 5; Sweden: two studies, *n* = 855), North America *n* = 32 studies with 355,862 participants (USA: 29 studies, *n* = 355,192; Canada: 3 studies, *n* = 670), South and Latin America *n* = 2 studies with 322 participants (both Brazil), Asia: *n* = 4 studies with 2,331 participants (India: two studies, *n* = 252; Japan: one study *n* = 177; Taiwan: 1 study, *n* = 1,902), Middle East and North Africa *n* = 10 studies with 4,361 participants (Israel: one study, *n* = 85; Saudi Arabia: one study, *n* = 117; Turkey: 8 studies, *n* = 4,159), Sub-Saharan Africa *n* = 2 studies with 737 participants (Nigeria: one study, *n* = 361; South Africa: one study, *n* = 376), Oceania *n* = 3 studies with 2,297 participants (all Australia), and one multinational study involving 144 English-speaking participants. Core sociodemographic criteria were often not fully reported or corrected for (especially regarding race/ethnicity, cigarette smoking, urbanity, and socio-economic status).

As described above, Viswasam and colleagues reported a PD prevalence of 3% (95%CI: 2–6%) during pregnancy, while Goodmann and colleagues found a prevalence rate of 1.7% (95%CI: 0.1–2.8%) postpartum. Further studies not included in these meta-analyses reported prevalence during pregnancy ranging from 1.1% [114] to 16.2% [123] and from 1.6% [31] to 3% [37] postpartum. Course and severity of PD were assessed in 11 prospective longitudinal studies. Outcomes were heterogeneous (showing improvement, worsening, or no change in PD symptoms) and associated with biological, psychological, and social factors (Table 2). A peripartum diagnosis of PD emerged as a risk factor for mental and somatic problems and disorders in both, the (expectant) mothers and their fetuses or infants (Tables 3 and 4). Parent-infant interaction in affected mother-infant dyads was adversely and persistently associated to the subsequent familiar development (e.g., maternal personal development and relationships) (Table 5).

**Table 2:** Risk factors, Course, and prevalence of panic disorder

Study, Country	Sample (peripartum women, if not specified otherwise)	Design	Measures and statistics	Assessments	Results
Aydogan et al. [6] *Turkey*	38 postpartum women with prior PD diagnosis	prospective	SCID (DSM-IV), PAS, HADS, COPE, MSPSS, TEMPS-A t test, Χ^2^ test, logistic regression	First day postpartum, 6–8 weeks postpartum	- Severity of panic symptoms decreased postpartum for a majority of patients (63%) and no changes (37%) were observed otherwise.
Benjamin [11] *USA*	1	case report	medical records	n/a	- First panic attack occurred during oxytocin challenge test in pregnancy.
Cohen et al. [17] *USA*	1	case report	medical records	n/a	- Amelioration of PD during pregnancy, placental abruption and life birth during severe panic attack around term.
Cohen et al. [18] *USA*	40	retrospective	medical records (DSM-III-R diagnosis, CGI) descriptive, Χ^2^ test	postpartum	- Patients with more severe PD did report a worsening in symptoms less frequently compared to mildly affected cases. - Most of the cases with deterioration in severity were not treated pharmacologically prior to birth.
Cohen et al. [19] *USA*	49	retrospective	medical records (DSM-III-R diagnosis, CGI) descriptive	postpartum	- No status change or improvement of PD in 38 women (78%), deterioration in 10 women (20%) during pregnancy. - Women with more (vs. less) severe panic symptoms received pharmacotherapy. Need for pharmaco-treatment after pregnancy did not differ significantly by group.
Cohen et al. [20] *USA*	10	prospective	SCID (DSM-III-R), CGI descriptive	throughout pregnancy (once every trimester) and postpartum (1–3-6–9 months)	- No changes in PD symptoms throughout the study. - Individual pharmacological treatment was maintained and intensified postpartum.
Curran et al. [26] *Ireland*	1	case report	medical records	n/a	- Full recovery of a PD patient during pregnancy, followed by relapse during the postpartum period.
Dannon et al. [27] *Israel*	68 (34 pregnant women, 34 non-pregnant controls)	prospective	medical records (PD DSM-IV diagnosis), PSQ t test, Χ^2^ test, ANOVA with repeated measures	baseline, every 4 weeks for 12 months, every 3 months for 6 years	- Patients who first experienced PD at the time of pregnancy had a higher risk of relapse (18/34) as compared with the PD-NP patients (12/34) over the course of the study. - Controlled and comparable treatment regimens (SSRI: paroxetine) were chosen for all participants.
Fairbrother et al. [31] *Canada*	310 (152 pregnant women above screening cutoffs)	prospective	screening instruments (GAD-7, OCI-R, Mini-SPIN, PDSR, MI, SPQ, PCL, EPDS), SCID (DSM-IV) descriptive	initial screening at 6–8 weeks postpartum, diagnostic interview 13 weeks postpartum	- PD prevalence during pregnancy and equally postpartum of 1.6%.
Gedam and Deka [37] *India*	100	cross sectional (convenience sample)	MINI Plus, HRSD, BPRS t test, Χ^2^ test	first 6 weeks postpartum	- PD point prevalence 3%.
George et al. [38] *USA*	3	case report	medical records	n/a	- Amelioration of PD during pregnancy.
Griez et al. [42] *Netherlands*	1	case report	medical records	n/a	- PD symptoms improved to remission in early pregnancy with a relapse 2 weeks prior to delivery. In a second pregnancy shortly after, the pattern was repeated.
Guler et al. [44] *Turkey*	13	prospective	SCID (DSM-IV) Wilcoxon signed rank test, U test	third trimester, 6 weeks postpartum	- PD severity decreased postpartum, for both pre-pregnancy and pregnancy onset patient groups.
Guler et al. [46] *Turkey*	1475	cross sectional (convenience sample)	SCID (DSM-IV) t test, Fisher’s exact test, Χ^2^ test, logistic regression	6.7% first, 23.3% second and 70% third trimester	- Incidence rate during pregnancy 1.3%. - Women with POPD were more likely than the controls to have a cluster C Axis II disorder and a history of a pre-existing anxiety or mood disorder.
Hendrick and Altshuler [48] *USA*	1	case report	medical records	n/a	- Patient with PD onset postpartum and worsening of symptoms during subsequent pregnancy, despite non- and pharmacological treatment.
Klein et al. [57] *USA*	27	retrospective longitudinal	medical records, SADS-LA t test, repeated measures ANOVA	records throughout pregnancy, interview ≥ 12 months postpartum	− 25/27 patients displayed amelioration of PD during pregnancy with relapse in postpartum period. - Lactation was associated with a mean panic level reduction for lactators versus nonlactators by 86% in the immediate months following delivery.
Kumar et al. [58] *India*	152	cross sectional (convenience sample)	MINI (DSM-IV) t test, Χ^2^ test	≤ 4 weeks postpartum	- PD point prevalence 2%.
Martini et al. [71], Martini et al. [74] *Germany*	306	prospective	CIDI-V (DSM-IV/ICD-10), obstetric records, measures of social support (F-Sozu), partnership quality (PFB) t test, Χ^2^ test	first trimester (baseline, lifetime assessment of PD), second trimester, third trimester as well as 10 days, 2 months, 4 months and 16 months postpartum	- PD prevalence in first trimester: 2.3%. - PD prevalence in second trimester: 5.1%. - PD prevalence in third trimester: 0.4%. - PD prevalence in 2 months postpartum: 1.1%. - PD prevalence in 4 months postpartum: 0%. - PD prevalence in 16 months postpartum: 1.5%. - Peripartum PD was associated with a worse psychosocial situation (reductions in social support and partnership quality).
Matthey et al. [75] *Australia*	764 (408 women, 356 men)	prospective	screening: EPDS, CES-D, Profile of Mood States; DIS (DSM-IV) Χ^2^ test	antenatal screening, diagnostic interview 6–8 weeks postpartum	- Maternal PD prevalence postpartum 1.7%. Paternal PD prevalence 0.5%. - Of women who reported a lifetime diagnosis of any anxiety disorder, 65.6% developed either postpartum anxiety or mood disorder vs. 29.4% of women with a history of depressive disorder.
Mauri et al. [76] *Italy*	1066	prospective	screening: EPDS, PDPI-R, WSAS; SCID (DSM-IV) logistic regression	first trimester: SCID, EPDS, PDPI-R, WSAS; EPDS at 1–3-6–9-12 months postpartum (with additional SCID when ≥ 13)	- PD peripartum prevalence 4%. - PD was associated both with depressive symptoms (RR = 5.27) and with MDD (RR = 7.23) in the first month postpartum, and predicted MDD during the first year postpartum (RR = 3.1).
Melville et al. [77] *USA*	1888	prospective	PHQ-15 (DSM-IV), medical records logistic regression	during pregnancy (gw ≥ 12), antenatal from medical records	- PD prevalence 3.2%.
Meshberg-Cohen and Svikis [78] *USA*	551 (412 pregnant women, 157 non-pregnant controls)	cross sectional (convenience sample)	PHQ (DSM-IV), STAI, AUQ, PAL t test, Χ^2^ test with Bonferroni correction, regression/correlation analysis	any point in pregnancy (first contact with OB/GYN services)	- PD point prevalence of non-pregnant women elevated (9.8%) vs. pregnant women (3.4%). - PD was associated with higher alcohol intake in all women (17.3 drinks/m vs. 3.1 drinks/m), though not in pregnant ones.
Nardi et al. (2003) *Brazil*	1	case report	medical records	not stated	- PD with prominent respiratory nocturnal panic attacks during pregnancy only
Pfeffer et al. [89] *Germany*	40	cross sectional (at-risk sample, peri-partum cardiomyopathy patients)	SCID (DSM-IV), BDI-II, WHOQoL t test, Χ^2^ test, U test	third trimester	- PD point prevalence 10%.
Uguz et al. [112] *Turkey*	1482 (1154 pregnant women, 328 non-pregnant controls)	case control (convenience sample)	SCID (DSM-IV) t test, Χ^2^ test, Fisher’s exact test	between 36th gestational week to 6 weeks postpartum	- PD prevalence during pregnancy 4.4% vs. 1.5% for non-pregnant controls (OR = 2.9).
Üstündağ Budak et al. [113] *multinational* (English speaking)	144 (67 cases with birth trauma, 77 controls with difficult birth but healthy infant)	case control (convenience sample, online)	PDSQ, EPDS, individualized delivery experience questionnaire ANOVA	maximum 4 years postpartum	- Birth loss was not associated with a diagnosis of PD.
Usuda et al. [114] *Japan*	177	cross sectional (convenience sample)	MINI (DSM-IV) descriptive	second trimester	- PD point prevalence 1.1%.
van Heyningen et al. [117] *South Africa*	376	cross sectional (convenience sample)	MINI (DSM-IV), MSPSS Fisher’s exact test, U test, logistic regression	any point in pregnancy (first contact with OB/GYN services)	- PD point prevalence 3%. - Any anxiety disorder was predicted by a history of mental health problems (OR = 4.11), major depressive episode (OR = 3.83), multigravidity (OR = 2.87), food insecurity (OR = 2.57), unplanned and unwanted pregnancy (OR = 2.14), pregnancy loss (OR = 2.1), and experience of threatening life events (OR = 1.3). Increased perceived social support reduced the risk for antenatal anxiety (OR = 0.95).
Verburg et al. [118] *Netherlands*	3 (with a total of 4 pregnancies)	case series	medical records	n/a	- In 3 pregnancies panic symptoms improved initially, but worsened in the second half. One patient developed PD in the second half of her pregnancy.
Viswasam et al. [123] *Australia*	200	prospective	MINI (DSM-5) descriptive, McNemar’s test	first, second and third trimester: MINI	- PD prevalence during pregnancy 16.2%. - One third experienced onset of PD during pregnancy.
Wenzel et al. [127] *USA*	788	cross sectional (at-risk sample, positive screening for dysphoria)	Screening: IDD, diagnosis: SCID (DSM-III) descriptive	4–7 months postpartum	- PD point prevalence 1.5%. Subclinical panic symptoms 11%. - The most common panic symptoms included heart palpitations, sweating, trembling, and paresthesia.
Wisner et al. [129] *USA*	1889 (168 women with CBROI, 1721 with NCBROI)	case control (at-risk sample from psychiatric records)	medical records Χ^2^ test, Fisher’s exact test	throughout pregnancy and postpartum period	- No differences in PD prevalence between cases (15%) vs. controls (11%). - If onset of any illness was related to child bearing, then to the postpartum period rather than the pregnancy.
Wisner et al. [130] *USA*	22 (women with PD and 45 pregnancies among them)	retrospective longitudinal	medical records, SADS-LA descriptive	throughout pregnancy and postpartum period, SADS 5 years postpartum	- Most PD patients experienced no change of their symptoms during and after pregnancy (49%), with consistency over multiple pregnancies only for 36% of multiparas. First lifetime onset of PD occurred postpartum or post-miscarriage in 13%.
Woods et al. [132] *USA*	1522	cross sectional (registry based)	medical records, PHQ-15 (DSM-IV), PPPSS t test, Χ^2^ test, logistic regression	second and third trimester (on average 23rd gestational week)	- PD prevalence 3.1%. - PD (OR = 6.8) was associated with high psychosocial stress.
Woolhouse et al. [133] *Australia*	1507	prospective	EPDS with additional questions on help seeking descriptive, Relative Risks	baseline: second trimester, follow-ups: third trimester, 3–6-9–12-18 months postpartum	- Subsyndromal panic and anxiety symptoms (in 8.5% of patients) reduced the likelihood of professional help seeking at any point in the study (RR = 0.68).

Comprehensively summarized in two systematic reviews/meta-analyses [40, 122] and thus excluded from our review of peripartum PD: Adewuya et al. [3], Farias et al. [33], Giardinelli et al. [35], Guler et al. [45], Marchesi et al. [68, 69], [103], Uguz et al. [109], Wenzel et al. (2005), Zar et al. (2002). See Supplemental Table 1 for further information

Regarding Mauri et al. [76]; conflicting outcome measures reported in original work, the more conservative is shown here

*ADIS* Anxiety Disorder Interview Schedule, *ANOVA* Analysis of Variance, *AUQ* Alcohol Use Questionnaire, *BAI* Beck Anxiety Inventory, *BDI-II* Beck Depression Inventory V.2, *BPRS* Brief Psychiatric Rating Scale, *CES-D* Centre for Epidemiological Studies Depression Scale, *DIS* Diagnostic Interview Schedule, *DAS* Dyadic Adjustment Scale, *DSM* Diagnostic and Statistical Manual of Mental Disorders, *EPDS* Edinburgh Postnatal Depression Scale, *FHS* Family History Screen, *F-Sozu* Social Support Questionnaire, *GAD-7* Generalized Anxiety Disorder Scale 7, *HRSD* Hamilton Rating Scale for Depression, *IDD* Inventory to Diagnose Depression, *INC* pregnancy onset PD, *MDD* Major Depressive Disorder, *MI* Mobility Inventory for Agoraphobia, *MINI* Mini-International Neuropsychiatric Interview, *Mini-SPIN* Mini Social Phobia Inventory, *MSPSS* Multidimensional Scale of Perceived Social Support, *(N)CBROI* (Non-)Childbearing Related Onset of Illness, *OB/GYN* Obstetrics and Gynecology, *OCI-R* Obsessive Compulsive Inventory – Revised, *PAL* Pregnancy Assessment of Lifestyle, *PCL* PTSD Checklist, *PD* Panic Disorder, *PDPI-R* Post-partum Depression Predictors Inventory-Revised, *PDSR* Panic Disorder Self-Report, *PPD* Postpartum Depression, *PPPSS* Prenatal Psychosocial Profile stress scale, *PSQ* Panic Self Questionnaire, *PSWQ* Penn State Worry Questionnaire, *PTSD* Post-Traumatic Stress Disorder, *SADS-LA* Schedule for Affective Disorders and Schizophrenia (-Lifetime Version modified for the study of anxiety disorders), *SCID* Structured Clinical Interview for the DSM, *SIAS* Social Interaction Anxiety Scale, *SPQ* Specific Phobia Questionnaire, *STAI* State-Trait Anxiety Inventory, *t test* Student’s t test for continuous variables, *U test* Mann-Whitney U test for non-parametric variables, *W-DEQ* Wijmer Delivery Expectancy/Experience Questionnaire, *WHOQoL* World Health Organization Quality of Life Questionnaire, *WSAS* Work and Social Adjustment Scale, *Χ*^*2*^ *test* Chi Square test for categorical variables

**Table 3:** Maternal health and birth outcomes

Study, Country	Sample	Design	Measures and Statistics	Assessments	Results
Bánhidy et al. [9] *Hungary*	38,151 pregnant women (187 cases with PD, 37964 controls)	case control (registry based)	medical records t and Χ^2^ test, prevalence odds ratios	throughout pregnancy and postpartum period (full clinical record)	- PD cases had reduced gestational age (0.4 weeks) and a larger proportion of preterm births (OR = 1.9). PD was associated with increased risk for polyhydramnios (OR = 3.3) and anemia (OR = 1.6). - No elevated risks were reported in mothers in pharmaco-treatment for PD.
Chen et al. [14] *Taiwan*	1902 pregnant women (317 cases with PD, 1585 matched controls)	case control (registry based)	health insurance data Χ^2^ test, logistic regression	throughout pregnancy and postpartum period (full insurance record)	- Treatment for panic attacks during pregnancy was associated with an increased risk of preterm birth (OR = 2.54) and SGA (OR = 2.29). - A prior diagnosis of PD without panic symptoms during pregnancy was associated with an increased risk of SGA (OR = 1.45).
Ciesielski et al. [15] *USA*	449	cross sectional (convenience sample)	medical records, genome-wide methylation profile U Test, Χ^2^ and Fisher’s Exact test, logistic regression	throughout pregnancy and postpartum period (full clinical record)	- Maternal prenatal mental disorder (depression, anxiety, OCD/panic) was associated with an increased risk of poor infant fetal growth (OR = 3.36): anxiety (OR = 3.15), OCD/PD (OR = 8.23). - Placental methylation patterns suggest a role for Leptin at a specific receptor site for SGA.
Cohen et al. [16] *Canada*	200	cross sectional (convenience sample)	EPDS, CTS, AAS Χ^2^ test, logistic regression	8–10 weeks postpartum	- Having a panic attack during pregnancy (OR = 5.4) was associated with an increased risk of PPD.
Goodwin et al. [41] *USA*	1517	cross sectional (representative national survey)	AUDADIS (DSM-IV) logistic regression	post or during pregnancy (pregnancy in the year before study)	- Nicotine dependence during pregnancy was associated with an increased risk of PD (OR = 3.1).
Johansen et al. [52] *USA*	336,522	cross sectional (insurance based)	ICD-9/10 diagnoses (insurance claims) logistic regression	throughout pregnancy and postpartum period (full insurance record)	- PD conferred risk for postpartum depression, independent of a comorbid depression history (OR 1.27, 2 months post-partum; OR 1.28, 1 year postpartum).
Kakaraparthi et al. [54] *Saudi Arabia*	117	prospective	medical records, HADS, GAD-7, PSS, BMWS t test, linear regression	any point during pregnancy	- The onset of the Covid-19 Pandemic coincided with a worsening in reported anxiety and depression, a diagnosis of PD predicted those changes.
Karjane et al. [53] *USA*	89	cross sectional (at-risk sample)	SCID (DSM-III-R), FADS t test, U test, Χ^2^ test, logistic regression	first trimester	- PD was more prevalent (15.4% vs. 0%) in a subgroup with a history of infertility in the context of alcohol abuse.
Kimmel et al. [55] *Sweden*	126	prospective	MINI (DSM-IV), STAI, EPDS, non-invasive heart rate U test, Fisher’s exact test, linear regressions with Bonferroni correction	second and third trimester, 6 weeks, 6 and 12 months postpartum	- Elevated lower heart frequency averages were found in PD patients during pregnancy, indicating higher sympathetic activation.
Magtira et al. [66] *USA*	108	cross sectional (convenience online sample)	self-report, medical records t test, Χ^2^ test	early postpartum	- There were no differences in pre-existing PD between recurring HG and non-recurring HG groups.
Martini et al. [71], Martini et al. [74] *Germany*	306	prospective	CIDI-V (DSM-IV/ICD-10), obstetric records t test, Χ^2^ test	first trimester: baseline, follow-ups: second and third trimester, 10 days, 2–4-16 months postpartum	- Infants of affected mothers presented with a lower birth weight (3011 g vs. 3381 g) and younger gestational age (38.4 weeks vs. 39.5 weeks) compared to controls.
Newport et al. [82] *USA*	686	case control	SCID (DMS-IV), HDRS, HARS and BDI, medical history taken from obstetric records t test, Χ^2^ test, logistic regression	throughout pregnancy and postpartum	- A lifetime PD diagnosis increased the risk for hypertensive disorders in pregnancy (OR = 1.78).
Ossola et al. [85] *Italy*	324	prospective	DSM-IV: PRIME-MD and HADS linear regression	first trimester: baseline, on average 5 follow-ups in pregnancy	- No effect of PD on birth weight, though the “Amount of Anxiety” (Area under Curve) reduces birth weight by 5.76 g per point.
Rambelli et al. [91] *Italy*	600	prospective	SCID (DSM-IV), EPDS, PDPI-R, FHS logistic regression	first trimester: full baseline, screening (EPDS) at 1–3-6 months postpartum	- PD during pregnancy (RR = 4.25), lifetime PD (RR = 2.47) and family history for PD (RR = 2.10) increased risk for PPD.
Rizzo et al. [95] *Italy*	133	prospective	GSM-V, EPDS Pearson correlation	third trimester: GSM-V, 3 & 6 months postpartum: EPDS	- Subthreshold panic symptoms during pregnancy were correlated to more postnatal depressive symptoms at 3 & 6 months postpartum.
Rogal et al. [97] *USA*	1339	prospective	MINI (DSM-IV), PHQ, self-report on substance use, obstetric and birth outcomes from medical records t test, Χ^2^ test, logistic regression	any point during pregnancy, postpartum data from medical records	- PD had no influence on birth weight or premature delivery. - PD was associated with a diagnosis of PTSD.
Sutter-Dallay et al. [105] *France*	945	prospective	MINI (DSM-IV), EPDS, DAS t test, Χ^2^ test, logistic regression	third trimester: baseline, follow-up at 6 weeks postpartum	- PD prevalence at baseline 1.4%. - Women with any pregnancy AD were more likely to present with intense postnatal depressive symptoms (EPDS ≥ 13, OR = 2.7).
Uguz et al. [110] *Turkey*	90 (19 pregnant women with PD, 24 with MDD, 22 GAD, 25 healthy controls)	case control (at-risk sample, perinatal mental health patients)	SCID (DSM-IV), child outcome from medical records Χ^2^ test, ANOVA, Cohen’s d, Tukey’s honestly significant difference	between 36th gestational week to 6 weeks postpartum	- Birth weight was lower in PD patients (2720 g) vs. all other groups (e.g. 3408 g for healthy controls). Gestational age was reduced (37.1 weeks) vs. healthy controls (39.6 weeks) and GAD patients (38.5 weeks).
Uguz et al. [111] *Turkey*	146 (44 pregnant women with PD in pharmaco-treatment, 52 with untreated PD, 50 healthy controls)	case control (at-risk sample, perinatal mental health patients)	SCID (DSM-IV), child outcome from medical records Χ^2^ test, Fisher’s exact test, ANOVA, Cohen’s d, Tukey’s honestly significant difference	between 36th gestational week to 6 weeks postpartum	- Pharmaco-treatment reduced adverse birth outcomes: untreated PD patients had infants with reduced gestational age (37.5 weeks) and lower birth weight (2809 g) and required more frequently neonatal care (25%) vs. treatment and healthy controls, which did not differ (39.1 weeks and 39.3 weeks, 3344 g and 3396 g, 9.1% and 4%).
Warren et al. [125] *USA*	58 dyads (25 mothers with PD, 33 healthy controls)	cross sectional (part of Warren et al. [124]	SCID (DSM-IV), birth outcomes taken from clinical records t test, U test, Fisher’s exact test, Χ^2^ test, linear regression	4–14 months postpartum	- Infants with PD mothers showed smaller birth weight (3338 g vs. 4445 g, β=−0.86) but were not significantly more likely to be born prematurely or earlier than controls (both 39.8 weeks).
Yonkers et al. [134] *USA*	2793 (98 pregnant women with PD)	prospective	WMH-CIDI, PTSD module MINI, EPDS, infant outcomes, and additional medical data from medical records logistic regression	baseline: first trimester, follow-ups: second to third trimester	- PD prevalence in pregnancy 3.7%. - After controlling for confounders, PD was not associated with maternal (hypertensive diseases of pregnancy, C-Section) or neonatal outcomes (birth weight, preterm birth, respiratory support). - Maternal benzodiazepine use was associated with cesarean delivery (OR = 2.45), low birth weight (OR = 3.41), and use of ventilatory support for the newborn (OR = 2.85). Maternal SSRI use was associated with hypertensive diseases of pregnancy (OR = 2.82), preterm birth (OR = 1.56), and use of minor respiratory interventions (OR = 1.81).

*AAS* Abuse Assessment Screen, *ANOVA* Analysis of Variance, *AUDADIS* Alcohol Abuse and Alcoholism Alcohol Use Disorder and Associated Disabilities Interview Schedule, *BDI* Beck Depression Inventory, *BMWS* Brief Measure of Worry Severity, *CTS* Conflict Tactics Scale, *DSM* Diagnostic and Statistical Manual of Mental Disorders, *EPDS* Edinburgh Postnatal Depression Scale, *FADS* Family Alcohol and Drug Survey, *GAD-7* Generalized Anxiety Disorder Scale 7, *GSM-V* General 5-Spectrum Measure, *HARS* Hamilton Anxiety Rating Scales, *HDRS* Hamilton Depression Rating Scale, *SGA* small-for-gestational-age, *HADS* Hospital Anxiety and Depression Scale, *HG* Hyperemesis gravidarum, *PD* Panic Disorder, *PPD* Postpartum Depression, *PSS* Perceived Stress Scale, *SCID* Structured Clinical Interview for the DSM, *t test* Student’s t test for continuous variables, *U test* Mann-Whitney U test for non-parametric variables, *WMH-CIDI* World Mental Health Composite International Diagnostic Interview, *Χ*^*2*^ *test* Chi Square test for categorical variables

**Table 4:** Infant Health

Study, Country	Sample	Design	Measure and Statistics	Assessments	Results
Ács et al. [2], Csáky-Szunyogh et al. [25] *Hungary*	60,994 pregnant women and their infants (22843 cases, 38151 controls)	case control (registry based)	medical records t test and Χ^2^ test, prevalence odds ratios	throughout pregnancy and postpartum period (full clinical records)	- Infants with CBD were more likely (OR = 1.6) to have had mothers with PD during the study pregnancy. - PD was associated with a higher risk of hypoplastic left heart syndrome (OR = 4.88), cleft lip with or without cleft palate (OR = 3.4), and multiple CBD (OR = 3.0). - No elevated risks were reported in mothers in pharmaco-treatment for PD.
Vereczkey et al. [119] *Hungary*	58,315 pregnant women and their infants (3562 cases with CHD, 16602 with other CBD, 38151 without CBD)	case control (registry based)	medical records t test and Χ^2^ test, logistic regression	throughout pregnancy and postpartum period (full clinical records)	- PD was associated with higher risk of hypoplastic left heart (OR = 13.38), other congenital abnormalities of the Aorta (OR = 8.83) and left-sided obstructive defects (OR = 5.43).

*AD* anxiety disorder(s), *CBD* Congenital Birth Defect, *CHD* Congenital Heart Defect, *DIPS* Diagnostic Interview for Psychiatric Disorders, *DSM* Diagnostic and Statistical Manual of Mental Disorders, *K-SADS-E* children’s version Schedule for Affective Disorders and Schizophrenia – Epidemiologic version, *PAIT* Parenting Attitudes towards Infants and Toddlers, *PD* Panic Disorder, *SCID* Structured Clinical Interview for the DSM, *t test* Student’s t test for continuous variables, *Χ*^*2*^ *test* Chi Square test for categorical variables

**Table 5:** Mother-Infant dyadic development

Study, Country	Sample	Design	Measures and Statistics	Assessments	Results
Martini et al. [71], Martini et al. [74] *Germany*	306	prospective	CIDI-V (DSM-IV/ICD-10), Baby-DIPS, maternal bonding (PBQ), parenting style (PAIT), neuropsychological development, infant temperament (HR and BI), and attachment (SSP) t test, Χ^2^ test	first trimester: baseline, follow-ups: second and third trimester, 10 days, 2–4-16 months postpartum	- Infants of affected mothers presented with shorter duration of breastfeeding (7 months vs. 10 months) and more regulatory problems compared to controls: feeding problems (53.9% vs. 25.3%) and sleeping problems (24.2% vs. 10.8%). - Mothers with PD displayed more bonding and parenting problems. - Neurophysiology and infant temperament (HR, BI) did not differ between groups.
Miller et al. [80] *USA*	104 mother-infant dyads	prospective	screening: IDAS; IMAS, IDAS; child behavior: ASQ, ECBQ Χ^2^ test, t test, hierarchical multiple regression	third trimester: screening, 16 months postpartum: clinical interview and child behavior	- Only maternal panic symptoms during pregnancy (not postpartum) were related to poorer problem-solving abilities (β=−0.27) and reduced effortful control skills (β=−0.25) in infants. - Communication, motor skills, personal/social skills, and negative affectivity were not associated with panic symptoms.
Reck et al. [93] *Germany*	87 mother-infant dyads (17 with maternal PD, 22 with other anxiety disorders, 48 controls)	cross sectional (convenience sample)	SCID (DSM-IV), FFSF paradigm (ICEP-R) t-test, U test, Χ^2^ test, ANOVA	on average 4 months postpartum	- No differences in infant positive and maternal behavior in cases vs. controls. Infant negative behavior (protest in FFSF) was increased in cases.
Sandre et al. [101] *Canada*	58 mother-infant dyads	cross sectional (convenience sample)	IDAS-II, PCIRS-IA, EEG measures (Nc) Pearson’s r, AN(C)OVA for repeated measures	postpartum (26–31 weeks)	- Infants of mothers reporting greater panic symptoms exhibited a larger (i.e., more negative) Nc to fearful mother and stranger faces. - The Nc elicited by mother and stranger faces was not significantly associated with mother’s sensitivity, warmth, intrusiveness, or infant positive engagement with the mother.
Warren et al. [124] *USA*	87 mother-infant dyads	cross sectional (convenience sample)	SCID (DSM-IV), infant temperament (4 months HR and 14 months BI, IBQ), infant neurophysiology (salivary cortisol, sleep, e.g. by SHQ), parenting behavior (PS), and infant-parent relationship (SSP) Χ^2^ test, t test, U test, ANOVA, Pearson’s and Point-Biserial correlation coefficients	4 months: 25 cases of maternal PD, 24 controls 14 months: 27 cases and 17 controls	- Infants with PD-affected mothers did not show more high reactivity, behavioral inhibition, or ambivalent/resistant attachment but did demonstrate different neurophysiology (higher salivary cortisol and more disturbed sleep) than controls. - Mothers with PD also displayed less sensitivity toward their infants and reported parenting behaviors concerning infant sleep and discipline that have been associated with child problems.
Weinberg et al. [126] *USA*	94 mother-infant dyads (13 with maternal PD, 33 with maternal MDD, 48 controls)	prospective	SCID (DSM-III-R), FFSF (IRSS, MRSS) ANOVA, ω^2^, Cohen’s d	3 months postpartum	No differences between case and control groups.

*ANOVA* Analysis of Variance, *ASQ* Ages and Stages Questionnaire, *BI* Behavioral Inhibition, *CARE-Index* Child-Adult Relationship Experimental Index, *Cohen’s d* effect size measure, *DSM* Diagnostic and Statistical Manual of Mental Disorders, *ECBQ* Early Childhood Behavior Questionnaire, *EEG* Electroencephalogram, *FFSF* Face-To-Face-Still-Face, *HR* High Reactivity, *IBQ* Infant Behavior Questionnaire, *ICEP-R* Infant and Caregiver Engagement Phases Revised, *IDAS* Inventory of Depression and Anxiety Symptoms, *IMAS* Interview for Mood and Anxiety Disorders, *IRSS* Infant Regulatory Scoring System, *MRSS* Maternal Regulatory Scoring System, *Nc* Negative Central, *ω*^*2*^ Omega squared, effect size measure, *PBQ* Postpartum Bonding Questionnaire, *PCIRS* Parent-Child Interaction Rating Scales, *PD* Panic Disorder, *PFB* Partnership Questionnaire, *PS* Parenting Scale, *SCID* Structured Clinical Interview for the DSM, *SHQ* Sleep Habit Questionnaire, *SSP* Strange Situation Procedure, *t test* Student’s t test for continuous variables, *U test* Mann-Whitney U test for non-parametric variables, *Χ*^*2*^ *test* Chi Square test for categorical variables

### Risk factors and course of peripartum panic disorder (table 2)

Table 2gives an overview of studies on the course of PD during peripartum period. Several case reports published in the 1980s and − 90s on the onset and course of PD during the peripartum period described a “classical presentation” with an improvement during pregnancy (“protective effect”) and subsequent deterioration of panic symptoms postpartum [11, 17, 23, 26, 38, 42, 48, 118]. Further quantitative studies examined the course of peripartum PD, reporting more heterogeneous presentations [18, 20, 56, 129, 130]. Since then, larger studies with a greater proportion of prospective designs have been published, not only from North America and Western Europe, but from all over the world, including 2 case control, 9 cross sectional, and 11 prospective studies (see Table 2). These studies revealed a much more heterogeneous picture of the appearance and course of PD than initially described. For example, a Canadian study reported equal prevalence rates of PD during pregnancy and postpartum of 1.6% [31], while a Turkish study found an increased share of women with PD (OR = 2.9, 95%CI: 1.2–7.2) compared to non-pregnant controls [112]. A comprehensive longitudinal study of 306 (expectant) mothers with 6 assessments during and after pregnancy showed prevalence rates ranging from 0.4 to 5.1% during the first, second, and third trimesters of pregnancy and between 1.1% (2 months) and 1.5% (16 months) postpartum [74]. Subsyndromal panic symptoms (i.e. at least one panic attack in the past month) were reported by 8.5% [133] and 11% of women [127] during the peripartum period.

Interestingly, antenatal psychosocial stress (e.g., low social support and low partnership quality) was associated with a higher risk of PD in the peripartum period [74, 117, 132].

### The role of peripartum PD for maternal and infant outcomes (tables 3 and 4)

A total of 21 studies, almost exclusively drawn from European and North American populations (95%) and predominantly published in the last 20 years examined the associations between PD and maternal health during pregnancy predominantly in obstetric and psychiatric settings (see Tables 3 and 4).

This evidence suggests an increased risk of pregnancy and birth complications in mothers with PD. For example, the risk of hypertensive diseases of pregnancy (OR = 1.8, 95%CI: 1.1–8) was increased in pregnant women with PD [82] and heart rate variability measurements in pregnant patients with PD indicated higher levels of sympathetic activation [55]. In addition, pregnant women with PD were more likely to have anemia (OR = 1.6, 95%CI: 1.2–2.3) and polyhydramnios (OR = 3.3, 95%CI: 1–10.3) than controls [9].

Regarding birth outcomes, PD appeared to be associated with significant reductions in birth weight (380–1107 g) in infants of mothers with PD compared to infants of healthy controls [74, 125]. Maternal PD was also associated with reduced fetal growth (small-for-gestational-age and intra-uterine growth restriction) with odds ratios between 1.5 (95%CI: 1.1–2.0) [14] and 8.2 (95%CI: 1.7–39.3). Ciesielski et al. [15], the latter when grouped with a diagnosis of obsessive-compulsive disorder. Furthermore a shorter gestational age with a reduction of 0.4–1.6 weeks was reported [9, 111]. However, other studies found no association for birth weight [86, 97] and preterm birth [97, 125] with PD.

Peripartum PD is often accompanied by its pharmacological treatment: In a study by Yonkers and colleagues, medication of PD with selective serotonin reuptake inhibitors (SSRI) was specifically associated with hypertensive disorders of pregnancy, preterm birth, and use of minor respiratory interventions in the newborn [134]. A study by Chen and colleagues tentatively supports this claim, finding a higher risk for preterm birth and small-for-gestational-age in women with any treatment for PD during pregnancy in their insurance records, including (unspecified) medication [14], whereas other studies on pharmaceutical treatment for PD did not reveal an elevated obstetric risk [9, 111, 134]

Unambiguously, nicotine dependence was associated with a higher risk for PD in the peripartum period [41]. Only one study systematically accounted for smoking and found no significant associations between peripartum PD and adverse birth outcomes after controlling for nicotine use, underlining its averse effects on maternal and infant health [134].

Postpartum depression was observed more frequently in mothers with PD than in healthy controls: The odds ratios ranged from 1.3 (95%CI: 1.1–1.4) [52]to 2.7 (95%CI: 1.1–6.3) [105] and the relative risks ranged from 3.1 (95%CI: 1.2–8.2) [76] to 4.3 (95%CI: 1.5–12.2) [91]. Even subsyndromal panic symptoms (i.e. panic attacks in pregnancy) were positively correlated with depressive symptoms at 3 and 6 months postpartum [95] and associated with an increased risk of postpartum depression (OR = 5.4, 95%CI: 1.6–19.0) [16]. Finally, an increased proportion of mothers with PD reported a history of alcohol use and infertility [53], although causality could not be established in this study.

Intriguing findings from a large registry-based Hungarian cohort study suggest a strong association between maternal PD and congenital malformations: Ács, Csáky-Szunyogh, Vereczkey and colleagues investigated the role of various maternal diseases in the development of various congenital birth defects [2, 25, 119]. They reported that PD was associated with a higher risk for rare diseases such as hypoplastic left heart, congenital anomalies of the aorta, left-sided atresia, cleft lip with or without cleft palate, and multiple congenital birth defects. In these studies, subgroups receiving medication for PD did not show an increased risk of congenital malformations.

#### The role of peripartum PD for mother-infant dyadic outcomes (table 5)

A healthy childhood is defined not only by the absence of diseases in early childhood, such as those described above, but also depends on a functional, bidirectional relationship between parents and their offspring. Recent studies have begun to examine lasting changes in children’s behavior and neurobiology initiated in utero and responses to pathological maternal behavior due to PD using comprehensive behavioral observations. The 5 studies that primarily (and the 2 that did so among other outcomes) reported on attachment and early development were all conducted in Germany [71, 74, 93] or North America [80, 101, 124, 126] and included 736 mother-infant dyads, almost half of whom in a single study [71, 74].

These studies did not report associations between parental PD and behavioral alterations in the infant, such as high reactivity, behavioral inhibition, or attachment [74, 124] or different maternal behavior between PD cases and control groups in the Face-Face-Still-Face paradigm [93, 126]. Differences in infant neurophysiology, namely a higher saliva cortisol concentration as a biomarker for psychosocial stress [124] and altered infant EEG signals in reaction to maternal and stranger faces [101], have been reported in two studies. A reduction in breastfeeding duration (7 months vs. 10 months) was observed in mothers with PD compared to controls [74]. Another study reported an 86% reduction in panic scores for breastfeeding women with PD [56].

## Discussion

This systematic review examined maternal, obstetric, neonatal, infant, and mother-infant dyadic outcomes of peripartum PD. Taken together, included studies showed (1) heterogeneous courses of maternal PD in the peripartum period (2) associations of maternal PD with subsequent postpartum depression and birth complications (e.g. intra-uterine growth restriction and shorter gestational age), and (3) a potentially increased risk of congenital birth defects at least in some cases.

Prevalence of PD in pregnancy [122], the postpartum period [40] or both [96] was already comprehensively examined in three systematic reviews/meta-analyses. We searched for further or more recent original publications on the epidemiology of PD and listed all studies that were not considered by the above mentioned reviews [31, 37, 58, 75, 77, 78, 112, 114, 117, 123, 132]. Evidently, the results of these publications were mostly within the confidence interval of the above-mentioned meta-analyses (except for Viswasam et al. with a PD prevalence of 16.2% during pregnancy).

The included studies showed heterogeneous courses/trajectories of PD in the peripartum period (e.g. exacerbation or improvement). The findings are consistent with evidence presented elsewhere for the course of other anxiety disorders during the peripartum period [28]. The reported hypothesis of “protection” against PD symptoms during pregnancy was not confirmed. Instead, some studies reported aggravation of panic attacks during pregnancy that may be associated with hormonal changes (e.g., progesterone, cortisol) and pregnancy-related physical changes. It was hypothesized that the adjustment of the cardiovascular system during early pregnancy, and associated internal bodily sensations may serve as interoceptive stimuli that provoke panic attacks during this time [74]. However, factors that contribute to the development and peripartum course of PD cannot be identified based on the literature gathered for this systematic review.

Smith and colleagues reported that women with PD were more likely to receive treatment than other peripartum patients with a mental disorder [103] The medical rather than psychiatric presentation of PD, including differential diagnoses such as pulmonary embolism and myocardial infarction, may account for more thorough diagnosis. Unfortunately, such high diagnostic accuracy has not been replicated elsewhere [104, 120]. In addition, Woolhouse and colleagues warned that anxiety and panic symptoms reduce professional help-seeking among afflicted mothers, in many cases due to embarrassment or a lack of trust [133]. Overall, the dearth of representative prospective studies hinders the conclusive treatment recommendations for different trajectories of peripartum PD.

Another focus was the examination of peripartum PD as a risk factor for maternal and infant outcomes. Panic attacks and prenatal stress may result in exposure of the feto-placental unit to stress hormones [22, 100]. In line with this hypothesis, we found that gestational complications such as low birth weight and preterm birth have been associated with maternal PD in most studies. These signs of prematurity are in turn associated with poor long-term prospects for affected children [94]. The emergence of pharmacotherapy as a potential prevention for these adverse effects [9, 111, 134] is encouraging and has also been reported in relation to infant health. However, the risks of medical treatment and untreated PD to mother and fetus during pregnancy must be balanced accordingly on a case-to-case basis. Maternal PD and subclinical panic symptoms were associated with higher risk of postpartum depression, which is consistent with a prior history of psychiatric illness in general [43, 59, 106]. It must be noted that as potential confounder maternal smoking status has either not been assessed in the included studies or, when assessed, not reported in their results [77]. Thus, negative effects described here and elsewhere [107] on infant, obstetric, and maternal health outcomes may be due to the confounding role of detrimental use of nicotine and tobacco during pregnancy [1, 81].

Finally, peripartum PD was examined as a risk factor for mother-infant dyadic outcomes in 8 studies using comprehensive behavioral observations. The included studies did not identify an increase of insecure attachment in children of mothers with PD, which is a known risk factor for later anxiety disorders [21, 30, 57, 67]. Similarly, specific parenting behaviors that have been linked to higher levels of child anxiety (e.g. overcontrol/anger, less emotional warmth/sensitivity) [30, 92], have not been associated with maternal PD [124]. Infant regulatory disorders (e.g. excessive infant crying, feeding, and sleeping disorders), which may be considered as the earliest psychopathological adversities [47] and may be common in infants of mothers with a history of anxiety disorders [73, 88] have not been examined in detail in studies on peripartum PD so far. However, this might be due to insufficient statistical power for the subgroup of women with PD. Finally, the role of PD on duration and quality of breastfeeding has only been assessed by two studies [56, 74] and remains to be further elucidated, as breastfeeding is beneficial for a variety of maternal and infant health outcomes [29], and the dyadic relationship [62, 64]. Interestingly, one study examined the role of paternal PD [75]. The significance of (expectant) fathers for the family environment during gestation and puerperium needs further scientific attention [5, 128]. They contribute not only to the fetal genome, but may also pose a direct risk to maternal health, for example in the genesis of pre-eclampsia [34]. Whether or not these known paternal traits are associated with a history of paternal PD should be elucidated in the future. There may also be additional unknown effects of paternal PD on familial transmission.

Taken together, no clear picture can be drawn from the recent studies regarding dyadic outcomes. It is encouraging that 5 of the 8 studies included in our review were published in 2018 or more recently, which may reflect a renewed interest in dyadic outcomes in families affected by PD, given the long latency between previous publications. However, further research is needed given the potential role of peripartum PD in the familial transmission of anxiety disorders [12, 32, 50, 98, 115].

### Strengths and limitations

This is the first systematic review on maternal PD and pregnancy comprising recent evidence on risk factors and course of perinatal PD and its impact on maternal and infant health, as well as later parent-child interaction. PD causes a significant burden for both individuals [7, 131], and might exacerbate during the peripartum period. Gaps in our current understanding of peripartum PD pertain amongst others the role of hormonal alterations during pregnancy (e.g. progesterone, cortisol), pregnancy-related physical changes (e.g. adjustment of the cardiovascular system during pregnancy), and the adverse effects of maternal smoking. The heterogeneity of previous studies (often with a single study per outcome measure) did not allow for a meta-analytic approach and thus a critical interpretative review was conducted. Therefore, we did not rigorously assess all included studies for risk of bias, as would have been appropriate in a statistical synthesis. We focused on controlling for selected biases (publication, recall), as mentioned above.

### Outlook and recommendations

Given the adverse effects of peripartum PD there is an urgent need for further systematic research and improvement of treatment. The need for adequate therapeutic support is underlined by those studies that considered pharmacological treatment: the described adverse effects on maternal and infant health disappeared and significant differences between PD and control groups were no longer observed [2, 9, 111, 134]. Legitimate concerns about side effects such as impaired fetal growth, preterm birth, or postnatal neonatal withdrawal [60, 83] of commonly used medications such as benzodiazepines or SSRIs during pregnancy remain [14, 134], but must be weighed against the benefits of PD remission. The putative beneficial effects of psychotherapy on maternal and infant health have yet to be systematically investigated as a treatment option for peripartum PD, compared to or in combination with pharmacological treatment. We also emphasize the systematic consideration of socioeconomic status, race, ethnicity, urbanity/rurality, and smoking status among others as potential confounders associated with peripartum PD that are under-reported in our sample of studies. Regarding PD trajectories, birth cohort studies rarely investigate mental health in detail [13], but would be an ideal instrument to understand the interaction of pregnancy and PD. For example the consistent association of prenatal psychosocial stress and maternal PD [74, 117, 132] could be tested more rigorously in this way. In addition, the lack of any neuroscientific imaging studies on peripartum PD is a notable exception from the growing body of evidence. Together with molecular analyses that go above and beyond our current understanding [15, 63], brain imaging could open another valuable perspective on the development of PD around birth. Given that MRI scans in pregnancy are considered unethical due to potential adverse effects on the fetus [36] there are no published data on brain activity in pregnant women with PD. However, imaging studies in postpartum women and analysis of existing images acquired for necessary primary care could be used to probe potential structural or functional anomalies in women with PD in peripartum period [116].

The vigilant approach that most care providers take daily (at least in the shared experience of these reviewers) is most valuable in the peripartum period and in the context of PD.

## Conclusions

The heterogenous courses of PD during pregnancy and after delivery require comprehensive monitoring of affected women during the peripartum period. Adverse effects of PD on various maternal and infant outcomes – particularly lower infant birth weight, maternal postpartum depression, and dyadic problems (e.g., breastfeeding and regulatory disorders) – highlight the need for increased awareness of PD among primary and specialist care providers to achieve early identification and prompt initiation of treatment.

## Supplementary Information

Supplementary Material 1.

Supplementary Material 2.

## Data Availability

Data are available from the corresponding author on reasonable request.

## References

[1] Abraham M, Alramadhan S, Iniguez C, Duijts L, Jaddoe VWV, Dekker HT, den, et al. A systematic review of maternal smoking during pregnancy and fetal measurements with meta-analysis. PLoS ONE. 2017;12(2):e0170946. 10.1371/journal.pone.0170946. 10.1371/journal.pone.0170946 PMC5322900 28231292

[2] Ács N, Bánhidy F, Horváth-Puhó E, Czeizel AE. Maternal panic disorder and congenital abnormalities: a population-based case-control study. Birth Defects Res Part - Clin Mol Teratology. 2006;76(4):253–61. 10.1002/bdra.20250. 10.1002/bdra.20250 16583439

[3] Adewuya AO, Ola BA, Aloba OO, Mapayi BM. Anxiety disorders among Nigerian women in late pregnancy: A controlled study. Arch Womens Ment Health. 2006;9(6):325–8. 10.1007/s00737-006-0157-5. 10.1007/s00737-006-0157-5 17033737

[4] Alder J, Fink N, Bitzer J, Hösli I, Holzgreve W. Depression and anxiety during pregnancy: a risk factor for obstetric, fetal and neonatal outcome? A critical review of the literature. J Maternal-Fetal Neonatal Med. 2007;20(3):189–209. 10.1080/14767050701209560. 10.1080/14767050701209560 17437220

[5] Allport BS, Johnson S, Aqil A, Labrique AB, Nelson T, KC A, et al. Promoting father involvement for child and family health. Acad Pediatr. 2018;18(7):746–53. 10.1016/j.acap.2018.03.011. 29653255 10.1016/j.acap.2018.03.011

[6] Aydogan S, Uguz F, Yakut E, Bayman MG, Gezginc K. The course and clinical correlates of panic disorder during the postpartum period: A naturalistic observational study. Revista Brasileira De Psiquiatria (Sao Paulo, Brazil: 1999). 2020;43(1):6–11. 10.1590/1516-4446-2020-1050. 10.1590/1516-4446-2020-1050 PMC7861186 33111774

[7] Bandelow B, Michaelis S. Epidemiology of anxiety disorders in the 21st century. Dialog Clin Neurosci. 2015;17(3):327–35. 10.31887/DCNS.2015.17.3/bbandelow. 10.31887/DCNS.2015.17.3/bbandelow PMC4610617 26487813

[8] Bandelow B, Sojka F, Broocks A, Hajak G, Bleich S, Rüther E. Panic disorder during pregnancy and postpartum period. Eur Psychiatry. 2006;21(7):495–500. 10.1016/j.eurpsy.2005.11.005. 16529913 10.1016/j.eurpsy.2005.11.005

[9] Bánhidy F, Acs N, Puhó E, Czeizel AE. Association between maternal panic disorders and pregnancy complications and delivery outcomes. Eur J Obstet Gynecol Reproductive Biology. 2006;124(1):47–52. 10.1016/j.ejogrb.2005.04.013. 10.1016/j.ejogrb.2005.04.013 15946784

[10] Bendau A, Martini J, Asselmann EPROSPERO. 2022 CRD42022313806. 2022. https://www.crd.york.ac.uk/prospero/display_record.php?ID=CRD42022313806. Accessed 12 Jul 2023.

[11] Benjamin J, Benjamin M. Panic disorder masquerading as pre-eclampsia. European Journal of Obstetrics, Gynecology, and Reproductive Biology. 1993;51(1):81–82. 10.1016/0028-2243(93)90195-i. 10.1016/0028-2243(93)90195-i 8282146

[12] Biederman J, Hirshfeld-Becker DR, Rosenbaum JF, Hérot C, Friedman D, Snidman N, et al. Further evidence of association between behavioral inhibition and social anxiety in children. Am J Psychiatry. 2001;158(10):1673–9. 10.1176/appi.ajp.158.10.1673. 11579001 10.1176/appi.ajp.158.10.1673

[13] Canova C, Cantarutti A. Population-based birth cohort studies in epidemiology. Int J Environ Res Public Health. 2020. 10.3390/ijerph17155276. 10.3390/ijerph17155276 PMC7432312 32717778

[14] Chen Y-H, Lin H-C, Lee H-C. Pregnancy outcomes among women with panic disorder — do panic attacks during pregnancy matter? J Affect Disord. 2010;120(1):258–62. 10.1016/j.jad.2009.04.025. 19428119 10.1016/j.jad.2009.04.025

[15] Ciesielski TH, Marsit CJ, Williams SM. Maternal psychiatric disease and epigenetic evidence suggest a common biology for poor fetal growth. BMC Pregnancy Childbirth. 2015;15(1):192. 10.1186/s12884-015-0627-8. 26303856 10.1186/s12884-015-0627-8 PMC4548904

[16] Cohen LS, Rosenbaum JF, Heller VL. Panic attack-associated placental abruption: a case report. J Clin Psychiatry. 1989;50(7):266–7. 2738032

[17] Cohen LS, Sichel DA, Dimmock JA, Rosenbaum JF. Impact of pregnancy on panic disorder: a case series. J Clin Psychiatry. 1994a;55(7):284–8. 7915272

[18] Cohen LS, Sichel DA, Dimmock JA, Rosenbaum JF. Postpartum course in women with preexisting panic disorder. J Clin Psychiatry. 1994b;55(7):289–92. 7915273

[19] Cohen LS, Sichel DA, Faraone SV, Robertson LM, Dimmock JA, Rosenbaum JF. Course of panic disorder during pregnancy and the puerperium: a preliminary study. Biol Psychiatry. 1996;39(11):950–4. 10.1016/0006-3223(95)00300-2. 9162207 10.1016/0006-3223(95)00300-2

[20] Cohen MM, Schei B, Ansara D, Gallop R, Stuckless N, Stewart DE. A history of personal violence and postpartum depression: is there a link? Archives Women’s Mental Health. 2002;4(3):83–92. 10.1007/s007370200004.

[21] Colonnesi C, Draijer EM, Jan JM, Stams G, van der Bruggen CO, Bögels SM, Noom MJ. The relation between insecure attachment and child anxiety: a meta-analytic review. J Clin Child Adolesc Psychol. 2011;40(4):630–45. 10.1080/15374416.2011.581623. 21722034 10.1080/15374416.2011.581623

[22] Coussons-Read ME. Effects of prenatal stress on pregnancy and human development: mechanisms and pathways. Obstet Med. 2013;6(2):52–7. 10.1177/1753495X12473751. 27757157 10.1177/1753495X12473751 PMC5052760

[23] Cowley DS, Roy-Byrne PP. Panic disorder during pregnancy. J Psychosom Obstet Gynecol. 1989;10(3):193–210. 10.3109/01674828909016694.

[24] Crowe RR, Noyes R, Pauls DL, Slymen D. A family study of panic disorder. Arch Gen Psychiatry. 1983;40(10):1065–9. 10.1001/archpsyc.1983.01790090027004. 6625855 10.1001/archpsyc.1983.01790090027004

[25] Csáky-Szunyogh M, Vereczkey A, Kósa Z, Gerencsér B, Czeizel AE. Risk factors in the origin of congenital left-ventricular outflow-tract obstruction defects of the heart: a population-based case–control study. Pediatr Cardiol. 2014;35(1):108–20. 10.1007/s00246-013-0749-6. 23843102 10.1007/s00246-013-0749-6

[26] Curran S, Nelson TE, Rodgers RJ. Resolution of panic disorder during pregnancy. Ir J Psychol Med. 1995;12(3):107–8. 10.1017/S0790966700014543.

[27] Dannon PN, Iancu I, Lowengrub K, Grunhaus L, Kotler M. Recurrence of panic disorder during pregnancy: a 7-year naturalistic follow-up study. Clin Neuropharmacol. 2006;29(3):132–7. 10.1097/01.WNF.0000220821.73017.14. 16772811 10.1097/01.WNF.0000220821.73017.14

[28] de Lijster JM, Dierckx B, Utens EMWJ, Verhulst FC, Zieldorff C, Dieleman GC, Legerstee JS. The age of onset of anxiety disorders. Can J Psychiatry. 2017;62(4):237–46. 10.1177/0706743716640757. 27310233 10.1177/0706743716640757 PMC5407545

[29] Dennis C-L, Falah-Hassani K, Shiri R. Prevalence of antenatal and postnatal anxiety: systematic review and meta-analysis. Br J Psychiatry. 2017;210(5):315–23. 10.1192/bjp.bp.116.187179. 28302701 10.1192/bjp.bp.116.187179

[30] Dieterich CM, Felice JP, O’Sullivan E, Rasmussen KM. Breastfeeding and health outcomes for the mother-infant dyad. Pediatr Clin North Am. 2013;60(1):31–48. 10.1016/j.pcl.2012.09.010. 23178059 10.1016/j.pcl.2012.09.010 PMC3508512

[31] Drake KL, Ginsburg GS. Family factors in the development, treatment, and prevention of childhood anxiety disorders. Clin Child Fam Psychol Rev. 2012;15(2):144–62. 10.1007/s10567-011-0109-0. 22241071 10.1007/s10567-011-0109-0

[32] Fairbrother N, Janssen P, Antony MM, Tucker E, Young AH. Perinatal anxiety disorder prevalence and incidence. J Affect Disord. 2016;200:148–55. 10.1016/j.jad.2015.12.082. 27131505 10.1016/j.jad.2015.12.082

[33] Farias DR, Pinto de TJP, Teofilo MMA, Vilela AAF, Vaz dos JS, Nardi AE, Kac G. Prevalence of psychiatric disorders in the first trimester of pregnancy and factors associated with current suicide risk. Psychiat Res. 2013;210(3):962–8. 10.1016/j.psychres.2013.08.053. 10.1016/j.psychres.2013.08.053 24090486

[34] Fox NA, Snidman N, Haas SA, Degnan KA, Kagan J. The relation between reactivity at 4 months and behavioral inhibition in the second year: replication across three independent samples. Infancy. 2015;20(1):98–114. 10.1111/infa.12063. 25574156 10.1111/infa.12063 PMC4283938

[35] Giardinelli L, Innocenti A, Benni L, Stefanini MC, Lino G, Lunardi C, Svelto V, Afshar S, Bovani R, Castellini G, Faravelli C. Depression and anxiety in perinatal period: Prevalence and risk factors in an Italian sample. Arch Womens Ment Health. 2012;15(1):21–30. 10.1007/s00737-011-0249-8. 10.1007/s00737-011-0249-8 22205237

[36] Galaviz-Hernandez C, Sosa-Macias M, Teran E, Garcia-Ortiz JE, Lazalde-Ramos BP. Paternal determinants preeclampsia. Front Physiol. 2018;9:1870. 10.3389/fphys.2018.01870. 30666213 10.3389/fphys.2018.01870 PMC6330890

[37] Gatta G, Di Grezia G, Cuccurullo V, Sardu C, Iovino F, Comune R, et al. MRI in pregnancy and precision medicine: a review from literature. J Personalized Med. 2021. 10.3390/jpm12010009. 10.3390/jpm12010009 PMC8778056 35055324

[38] Gedam S, Deka K. Psychiatric morbidity in puerperium: incidence, associated socio-demographic and obstetric risk factors. 2014.

[39] George DT, Ladenheim JA, Nutt DJ. Effect of pregnancy on panic attacks. Am J Psychiatry. 1987;144(8):1078–9. 10.1176/ajp.144.8.1078. 3605431 10.1176/ajp.144.8.1078

[40] Godin K, Stapleton J, Kirkpatrick SI, Hanning RM, Leatherdale ST. Applying systematic review search methods to the grey literature: a case study examining guidelines for school-based breakfast programs in Canada. Syst Rev. 2015;4(1):138. 10.1186/s13643-015-0125-0. 26494010 10.1186/s13643-015-0125-0 PMC4619264

[41] Goodman JH, Watson GR, Stubbs B. Anxiety disorders in postpartum women: a systematic review and meta-analysis. J Affect Disord. 2016;203:292–331. 10.1016/j.jad.2016.05.033. 27317922 10.1016/j.jad.2016.05.033

[42] Goodwin RD, Keyes K, Simuro N. Mental disorders and nicotine dependence among pregnant women in the United States. Obstet Gynecol. 2007;109(4):875–83. 10.1097/01.AOG.0000255979.62280.e6. 17400849 10.1097/01.AOG.0000255979.62280.e6

[43] Griez EJL, Hauzer R, Meijer J. Pregnancy and estrogen-induced panic. Am J Psychiatry. 1995;152:1688. 10.1176/ajp.152.11.1688a. 7485637 10.1176/ajp.152.11.1688a

[44] Guler O, Koken GN, Emul M, Ozbulut O, Gecici O, Uguz F, Gezginc K, Zeytinci IE, Karatayli S, Askin R. Course of panic disorder during the early postpartum period: A prospective analysis. Compr Psychiatry. 2008a;49(1):30–4. 10.1016/j.comppsych.2007.06.007. 10.1016/j.comppsych.2007.06.007 18063038

[45] Guler O, Sahin FK, Emul HM, Ozbulut O, Gecici O, Uguz F, Gezginc K, Zeytinci IE, Karatayli S, Askin R. The prevalence of panic disorder in pregnant women during the third trimester of pregnancy. Comprehensive Psychiatry. 2008b;49(2):154–8. 10.1016/j.comppsych.2007.08.008. 10.1016/j.comppsych.2007.08.008 18243887

[46] Guler O, Kaya V, Gezginç K, Kayhan F, Çiçek E, Sönmez Ö, Uğuz F. Pregnancy-Onset Panic Disorder: Incidence, Comorbidity and Associated Factors. Noro Psikiyatri Arsivi. 2015;52(3):216–20. 10.5152/npa.2015.7565. 10.5152/npa.2015.7565 PMC5353051 28360713

[47] Guintivano J, Manuck T, Meltzer-Brody S. Predictors of postpartum depression: a comprehensive review of the last decade of evidence. Clin Obstet Gynecol. 2018;61(3):591–603. 10.1097/GRF.0000000000000368. 29596076 10.1097/GRF.0000000000000368 PMC6059965

[48] Hemmi MH, Wolke D, Schneider S. Associations between problems with crying, sleeping and/or feeding in infancy and long-term behavioural outcomes in childhood: a meta-analysis. Arch Dis Child. 2011;96(7):622–9. 10.1136/adc.2010.191312. 21508059 10.1136/adc.2010.191312

[49] Hendrick VC, Altshuler LL. Management of breakthrough panic disorder symptoms during pregnancy. J Clin Psychopharmacol. 1997;17(3):228–9. 10.1097/00004714-199706000-00019. 9169973 10.1097/00004714-199706000-00019

[50] Hertzberg T, Wahlbeck K. The impact of pregnancy and puerperium on panic disorder: a review. J Psychosom Obstet Gynecol. 1999;20(2):59–64. 10.3109/01674829909075578. 10.3109/01674829909075578 10422037

[51] Hirshfeld-Becker DR, Biederman J, Faraone SV, Robin JA, Friedman D, Rosenthal JM, Rosenbaum JF. Pregnancy complications associated with childhood anxiety disorders. Depress Anxiety. 2004;19(3):152–62. 10.1002/da.20007. 15129417 10.1002/da.20007

[52] Jacobi F, Höfler M, Strehle J, Mack S, Gerschler A, Scholl L, et al. Twelve-months prevalence of mental disorders in the German Health Interview and examination survey for adults - Mental Health Module (DEGS1-MH): a methodological addendum and correction. Int J Methods Psychiatr Res. 2015;24(4):305–13. 10.1002/mpr.1479. 26184561 10.1002/mpr.1479 PMC6878329

[53] Johansen SL, Stenhaug BA, Robakis TK, Williams KE, Cullen MR. Past psychiatric conditions as risk factors for postpartum depression: a nationwide cohort study. J Clin Psychiatry. 2020;81(1):E1–9. 10.4088/JCP.19m12929. 10.4088/JCP.19m12929 31967747

[54] Kakaraparthi VN, Alshahrani MS, Reddy RS, Samuel PS, Tedla JS, Dixit S, Gautam AP, Rengaramanujam K, Gular K, Kakaraparthi L, Ahmad I. Anxiety, depression, worry, and stress-related perceptions among antenatal women during the COVID-19 pandemic: Single group repeated measures design. Ind J Psychiat. 2022;64(1):64–72. 10.4103/indianjpsychiatry.indianjpsychiatry_1359_20. 10.4103/indianjpsychiatry.indianjpsychiatry_1359_20 PMC8992752 35400735

[55] Karjane NW, Stovall DW, Berger NG, Svikis DS. Alcohol abuse risk factors and psychiatric disorders in pregnant women with a history of infertility. J Women’s Health. 2008;17(10):1623–7. 10.1089/jwh.2007.0651. 10.1089/jwh.2007.0651 18710366

[56] Kimmel MC, Fransson E, Cunningham JL, Brann E, Grewen K, Boschiero D, et al. Heart rate variability in late pregnancy: exploration of distinctive patterns in relation to maternal mental health. Transl Psychiatry. 2021. 10.1038/s41398-021-01401-y. 10.1038/s41398-021-01401-y PMC8119957 33986246

[57] Klein DF, Skrobala AM, Garfunkel RS. Preliminary look at the effects of pregnancy on the course of panic disorder. Anxiety. 1994;1(5):227–32. 10.1002/anxi.3070010507. 9160579

[58] Kraft A, Knappe S, Petrowski K, Petzoldt J, Martini J. Unterschiede in der mutter-kind-bindung bei frauen mit und ohne soziale phobie. Z Kinder Jugendpsychiatr Psychother. 2017;45(1):49–57. 10.1024/1422-4917/a000454. 27428793 10.1024/1422-4917/a000454

[59] Kumar N, Nagaraj AKM, Koudike U, Majgi SM. Psychiatric morbidity and correlates in postpartum women in a tertiary care hospital. Indian J Psychol Med. 2016;38(4):309–14. 10.4103/0253-7176.185956. 27570341 10.4103/0253-7176.185956 PMC4980897

[60] Le Strat Y, Dubertret C, Le Foll B. Prevalence and correlates of major depressive episode in pregnant and postpartum women in the United States. J Affect Disord. 2011;135(1–3):128–38. 10.1016/j.jad.2011.07.004. 21802737 10.1016/j.jad.2011.07.004

[61] Levinson-Castiel R, Merlob P, Linder N, Sirota L, Klinger G. Neonatal abstinence syndrome after in utero exposure to selective serotonin reuptake inhibitors in term infants. Arch Pediatr Adolesc Med. 2006;160(2):173–6. 10.1001/archpedi.160.2.173. 16461873 10.1001/archpedi.160.2.173

[62] Linde K, Lehnig F, Nagl M, Kersting A. The association between breastfeeding and attachment: a systematic review. Midwifery. 2020;81:102592. 10.1016/j.midw.2019.102592. 31830673 10.1016/j.midw.2019.102592

[63] Litzky JF, Deyssenroth MA, Everson TM, Lester BM, Lambertini L, Chen J, Marsit CJ. Prenatal exposure to maternal depression and anxiety on imprinted gene expression in placenta and infant neurodevelopment and growth. Pediatr Res. 2018;83(5):1075–83. 10.1038/pr.2018.27. 29538358 10.1038/pr.2018.27 PMC5959758

[64] Liu J, Leung P, Yang A. Breastfeeding and active bonding protects against children’s internalizing behavior problems. Nutrients. 2013;6(1):76–89. 10.3390/nu6010076. 24368674 10.3390/nu6010076 PMC3916850

[65] Low NCP, Cui L, Merikangas KR. Specificity of familial transmission of anxiety and comorbid disorders. J Psychiatr Res. 2008;42(7):596–604. 10.1016/j.jpsychires.2007.07.002. 17706672 10.1016/j.jpsychires.2007.07.002

[66] Magtira A, Paik Schoenberg F, MacGibbon K, Tabsh K, Fejzo MS. Psychiatric factors do not affect recurrence risk of hyperemesis gravidarum. J Obstet Gynaecol Res. 2015;41(4):512–6. 10.1111/jog.12592. 10.1111/jog.12592 25345812

[67] Manassis K, Bradley S, Goldberg S, Hood J, Swinson RP. Behavioural inhibition, attachment and anxiety in children of mothers with anxiety disorders. Can J Psychiatry. 1995;40(2):87–92. 10.1177/070674379504000206. 7788623 10.1177/070674379504000206

[68] Marchesi C, Giaracuni G, Paraggio C, Ossola P, Tonna M, De Panfilis C. Pre-morbid alexithymia in panic disorder: A cohort study. Psychiat Res. 2014;215(1):141–5. 10.1016/j.psychres.2013.10.030. 10.1016/j.psychres.2013.10.030 24230995

[69] Marchesi C, Ampollini P, Paraggio C, Giaracuni G, Ossola P, de Panfilis C, et al. Risk factors for panic disorder in pregnancy: a cohort study. J Affect Disord. 2014;156:134–8. 10.1016/j.jad.2013.12.006. 24388039 10.1016/j.jad.2013.12.006

[70] Maron E, Hettema JM, Shlik J. Advances in molecular genetics of panic disorder. Mol Psychiatry. 2010;15(7):681–701. 10.1038/mp.2009.145. 20048750 10.1038/mp.2009.145

[71] Martini J, Wittich J, Petzoldt J, Winkel S, Einsle F, Siegert J, et al. Maternal anxiety disorders prior to conception, psychopathology during pregnancy and early infants’ development: a prospective-longitudinal study. Arch Womens Ment Health. 2013;16(6):549–60. 10.1007/s00737-013-0376-5. 24057868 10.1007/s00737-013-0376-5

[72] Martini J, Petzoldt J, Einsle F, Beesdo-Baum K, Höfler M, Wittchen HU. Risk factors and course patterns of anxiety and depressive disorders during pregnancy and after delivery: a prospective-longitudinal study. J Affect Disord. 2015;175:385–95. 10.1016/j.jad.2015.01.012. 25678171 10.1016/j.jad.2015.01.012

[73] Martini J, Petzoldt J, Knappe S, Garthus-Niegel S, Asselmann E, Wittchen HU. Infant, maternal, and familial predictors and correlates of regulatory problems in early infancy: the differential role of infant temperament and maternal anxiety and depression. Early Hum Dev. 2017;115:23–31. 10.1016/j.earlhumdev.2017.08.005. 28869923 10.1016/j.earlhumdev.2017.08.005

[74] Martini J, Beesdo-Baum K, Garthus-Niegel S, Wittchen HU. The course of panic disorder during the peripartum period and the risk for adverse child development: a prospective-longitudinal study. J Affect Disord. 2020;266:722–30. 10.1016/j.jad.2020.01.018. 32217255 10.1016/j.jad.2020.01.018

[75] Matthey S, Barnett B, Howie P, Kavanagh DJ. Diagnosing postpartum depression in mothers and fathers: whatever happened to anxiety? J Affect Disord. 2003;74(2):139–47. 10.1016/s0165-0327(02)00012-5. 12706515 10.1016/s0165-0327(02)00012-5

[76] Mauri M, Oppo A, Montagnani MS, Borri C, Banti S, Camilleri V, et al. Beyond postpartum depressions: specific anxiety diagnoses during pregnancy predict different outcomes: results from PND-ReScU. J Affect Disord. 2010;127(1):177–84. 10.1016/j.jad.2010.05.015. 20554326 10.1016/j.jad.2010.05.015

[77] Melville JL, Gavin A, Guo Y, Fan MY, Katon WJ. Depressive disorders during pregnancy: prevalence and risk factors in a large urban sample. Obstet Gynecol. 2010;116(5):1064–70. 10.1097/AOG.0b013e3181f60b0a. 20966690 10.1097/AOG.0b013e3181f60b0a PMC3068619

[78] Meshberg-Cohen S, Svikis D. Panic disorder, trait anxiety, and alcohol use in pregnant and nonpregnant women. Compr Psychiatr. 2007;48(6):504–10. 10.1016/j.comppsych.2007.06.004. 10.1016/j.comppsych.2007.06.004 17954134

[79] Micco JA, Henin A, Mick E, Kim S, Hopkins CA, Biederman J, Hirshfeld-Becker DR. Anxiety and depressive disorders in offspring at high risk for anxiety: a meta-analysis. J Anxiety Disord. 2009;23(8):1158–64. 10.1016/j.janxdis.2009.07.021. 19709850 10.1016/j.janxdis.2009.07.021

[80] Miller ML, Williams BM, McCabe JE, Williamson JA, King S, Laplante DP, et al. Perinatal anxiety and depressive symptoms and perception of child behavior and temperament in early motherhood. J Dev Origins Health Disease. 2021;12(3):513–22. 10.1017/S2040174420000781. 10.1017/S2040174420000781 32907691

[81] Mund M, Louwen F, Klingelhoefer D, Gerber A. Smoking and pregnancy–a review on the first major environmental risk factor of the unborn. Int J Environ Res Public Health. 2013;10(12):6485–99. 10.3390/ijerph10126485. 24351784 10.3390/ijerph10126485 PMC3881126

[82] Newport DJ, Hostetter AL, Juul SH, Porterfield SM, Knight BT, Stowe ZN. Prenatal psychostimulant and antidepressant exposure and risk of hypertensive disorders of pregnancy. J Clin Psychiatry. 2016;77(11):1538–45. 10.4088/JCP.15m10506. 28076672 10.4088/JCP.15m10506

[83] Nordeng H, Lindemann R, Perminov KV, Reikvam A. Neonatal withdrawal syndrome after in utero exposure to selective serotonin reuptake inhibitors. Acta Paediatr (Oslo Norway: 1992). 2001;90(3):288–91. 11332169

[84] Northcott CJ, Stein MB. Panic disorder in pregnancy. J Clin Psychiatry. 1994;55(12):539–42. 7814349

[85] Ossola P, Ampollini P, Gerra ML, Tonna M, Viviani D, Marchesi C. Anxiety, depression, and birth outcomes in a cohort of unmedicated women. The Journal of Maternal-Fetal & Neonatal Medicine. 2021;34(10):1606–12. 10.1080/14767058.2019.1641483. 10.1080/14767058.2019.1641483 31328591

[86] Ossola P, Ampollini P, Gerra ML, Tonna M, Viviani D, Marchesi C. Anxiety, depression, and birth outcomes in a cohort of unmedicated women. J Maternal-Fetal Neonatal Med. 2021;34(10):1606–12. 10.1080/14767058.2019.1641483. 10.1080/14767058.2019.1641483 31328591

[87] Page MJ, McKenzie JE, Bossuyt PM, Boutron I, Hoffmann TC, Mulrow CD, et al. The PRISMA 2020 statement: an updated guideline for reporting systematic reviews. BMJ. 2021;372:n71. 10.1136/bmj.n71. 33782057 10.1136/bmj.n71 PMC8005924

[88] Petzoldt J, Wittchen HU, Einsle F, Martini J. Maternal anxiety versus depressive disorders: specific relations to infants’ crying, feeding and sleeping problems. Child Care Health Dev. 2016;42(2):231–45. 10.1111/cch.12292. 26490836 10.1111/cch.12292

[89] Pfeffer TJ, Herrmann J, Berliner D, König T, Winter L, Ricke-Hoch M, Ponimaskin E, Schuchardt S, Thum T, Hilfiker-Kleiner D, Bauersachs J, Kahl KG. Assessment of major mental disorders in a German peripartum cardiomyopathy cohort. ESC Heart Failure. 2020;7(6):4394–8. 10.1002/ehf2.12967. 10.1002/ehf2.12967 PMC7754901 32909398

[90] Quagliato LA, de Matos UMA, Nardi AE. Lifetime psychopathology in the offspring of parents with anxiety disorders: a systematic review. J Affect Disord. 2022;319:618–26. 10.1016/j.jad.2022.09.049. 36174782 10.1016/j.jad.2022.09.049

[91] Rambelli C, Montagnani MS, Oppo A, Banti S, Borri C, Cortopassi C, et al. Panic disorder as a risk factor for post-partum depression: results from the Perinatal Depression-Research & Screening Unit (PND-ReScU) study. J Affect Disord. 2010;122(1):139–43. 10.1016/j.jad.2009.07.002. 19651446 10.1016/j.jad.2009.07.002

[92] Rapee RM. Family factors in the development and management of anxiety disorders. Clin Child Fam Psychol Rev. 2012;15(1):69–80. 10.1007/s10567-011-0106-3. 22116624 10.1007/s10567-011-0106-3

[93] Reck C, Tietz A, Müller M, Seibold K, Tronick E. The impact of maternal anxiety disorder on mother-infant interaction in the postpartum period. PLoS ONE. 2018;13(5):e0194763. 10.1371/journal.pone.0194763. 29799842 10.1371/journal.pone.0194763 PMC5969737

[94] Risnes KR, Vatten LJ, Baker JL, Jameson K, Sovio U, Kajantie E, et al. Birthweight and mortality in adulthood: a systematic review and meta-analysis. Int J Epidemiol. 2011;40(3):647–61. 10.1093/ije/dyq267. 21324938 10.1093/ije/dyq267

[95] Rizzo A, Bruno A, Torre G, Mento C, Pandolfo G, Cedro C, et al. Subthreshold psychiatric symptoms as potential predictors of postpartum depression. Health Care Women Int. 2022;43(1–3):129–41. 10.1080/07399332.2021.1963730. 34652261 10.1080/07399332.2021.1963730

[96] Roddy Mitchell A, Gordon H, Atkinson J, Lindquist A, Walker SP, Middleton A, et al. Prevalence of perinatal anxiety and related disorders in low- and middle-income countries: a systematic review and Meta-analysis. JAMA Netw open. 2023;6(11):e2343711. 10.1001/jamanetworkopen.2023.43711. 37976063 10.1001/jamanetworkopen.2023.43711 PMC10656650

[97] Rogal SS, Poschman K, Belanger K, Howell HB, Smith MV, Medina J, Yonkers KA. Effects of posttraumatic stress disorder on pregnancy outcomes. J Affect Disord. 2007;102(1):137–43. 10.1016/j.jad.2007.01.003. 17291588 10.1016/j.jad.2007.01.003 PMC2150994

[98] Rosenbaum JF, Biederman J, Gersten M, Hirshfeld DR, Meminger SR, Herman JB, et al. Behavioral inhibition in children of parents with panic disorder and agoraphobia. A controlled study. Arch Gen Psychiatry. 1988;45(5):463–70. 10.1001/archpsyc.1988.01800290083010. 3358645 10.1001/archpsyc.1988.01800290083010

[99] Rosenbaum JF, Biederman J, Hirshfeld-Becker DR, Kagan J, Snidman N, Friedman D, et al. A controlled study of behavioral inhibition in children of parents with panic disorder and depression. Am J Psychiatry. 2000;157(12):2002–10. 10.1176/appi.ajp.157.12.2002. 11097967 10.1176/appi.ajp.157.12.2002

[100] Sandman CA, Davis EP, Buss C, Glynn LM. Exposure to prenatal psychobiological stress exerts programming influences on the mother and her fetus. Neuroendocrinology. 2012;95(1):7–21. 10.1159/000327017. 21494029 10.1159/000327017 PMC7068789

[101] Sandre A, Freeman C, Renault H, Humphreys KL, Weinberg A. Maternal symptoms of depression and anxiety during the postpartum period moderate infants’ neural response to emotional faces of their mother and of female strangers. Cogn Affect Behav Neurosci. 2022;22(6):1370–89. 10.3758/s13415-022-01022-y. 35799031 10.3758/s13415-022-01022-y

[102] Sholomskas DE, Wickamaratne PJ, Dogolo L, O’Brien DW, Leaf PJ, Woods SW. Postpartum onset of panic disorder: a coincidental event? J Clin Psychiatry. 1993;54(12):476–80. 8276738

[103] Smith MV, Rosenheck RA, Cavaleri MA, Howell HB, Poschman K, Yonkers KA. Screening for and detection of depression, panic disorder, and PTSD in public-sector obstetric clinics. Psychiatric Serv (Washington D C). 2004;55(4):407–14. 10.1176/appi.ps.55.4.407. 10.1176/appi.ps.55.4.407 15067153

[104] Spitzer RL, Williams JB, Kroenke K, Hornyak R, McMurray J. Validity and utility of the PRIME-MD patient health questionnaire in assessment of 3000 obstetric-gynecologic patients: the PRIME-MD Patient Health Questionnaire obstetrics-Gynecology Study. Am J Obstet Gynecol. 2000;183(3):759–69. 10.1067/mob.2000.106580. 10992206 10.1067/mob.2000.106580

[105] Sutter-Dallay AL, Giaconne-Marcesche V, Glatigny-Dallay E, Verdoux H. Women with anxiety disorders during pregnancy are at increased risk of intense postnatal depressive symptoms: a prospective survey of the MATQUID cohort. Eur Psychiatr. 2004;19(8):459–63. 10.1016/j.eurpsy.2004.09.025. 10.1016/j.eurpsy.2004.09.025 15589703

[106] Tebeka S, Le Strat Y, Dubertret C. Developmental trajectories of pregnant and postpartum depression in an epidemiologic survey. J Affect Disord. 2016;203:62–8. 10.1016/j.jad.2016.05.058. 27280964 10.1016/j.jad.2016.05.058

[107] Teixeira JM, Fisk NM, Glover V. Association between maternal anxiety in pregnancy and increased uterine artery resistance index: cohort based study. BMJ. 1999;318(7177):153–7. 10.1136/bmj.318.7177.153. 9888905 10.1136/bmj.318.7177.153 PMC27690

[108] Telman LGE, van Steensel FJA, Maric M, Bögels SM. What are the odds of anxiety disorders running in families? A family study of anxiety disorders in mothers, fathers, and siblings of children with anxiety disorders. Eur Child Adolesc Psychiatry. 2018;27(5):615–24. 10.1007/s00787-017-1076-x. 29110074 10.1007/s00787-017-1076-x PMC5945734

[109] Uguz F, Gezginc K, Kayhan F, Sarı S, Büyüköz D. Is pregnancy associated with mood and anxiety disorders? A cross-sectional study. Gen Hosp Psychiatry. 2010;32(2):213–5. 10.1016/j.genhosppsych.2009.11.002. 10.1016/j.genhosppsych.2009.11.002 20302997

[110] Uguz F, Sahingoz M, Sonmez EO, Karsidag C, Yuksel G, Annagur BB, Annagur A. The effects of maternal major depression, generalized anxiety disorder, and panic disorder on birth weight and gestational age: A comparative study. J Psychosom Res. 2013;75(1):87–9. 10.1016/j.jpsychores.2013.02.008. 10.1016/j.jpsychores.2013.02.008 23751245

[111] Uguz F, Yuksel G, Onur OS, Karsidag C, Gezginc K, Arpaci N. Neonatal outcomes in pregnant women with untreated and treated panic disorder. Compr Psychiatr. 2018;87:107–11. 10.1016/j.comppsych.2018.10.001. 10.1016/j.comppsych.2018.10.001 30326358

[112] Uguz F, Yakut E, Aydogan S, Bayman MG, Gezginc K. Prevalence of mood and anxiety disorders during pregnancy: a case-control study with a large sample size. Psychiatry Res. 2019;272:316–8. 10.1016/j.psychres.2018.12.129. 30597383 10.1016/j.psychres.2018.12.129

[113] Üstündağ Budak AM, Harris G, Blissett J. Perinatal trauma with and without loss experiences. J Reprod Infant Psychol. 2016;34(4):413–25. 10.1080/02646838.2016.1186266.

[114] Usuda K, Nishi D, Makino M, Tachimori H, Matsuoka Y, Sano Y, et al. Prevalence and related factors of common mental disorders during pregnancy in Japan: a cross-sectional study. Biopsychosoc Med. 2016;10:17. 10.1186/s13030-016-0069-1. 27213012 10.1186/s13030-016-0069-1 PMC4874014

[115] van Brakel AML, Muris P, Bögels SM, Thomassen C. A multifactorial model for the etiology of anxiety in non-clinical adolescents: Main and Interactive effects of behavioral inhibition, attachment and parental rearing. J Child Fam Stud. 2006;15(5):568–78. 10.1007/s10826-006-9061-x.

[116] van den Heuvel MI, Thomason ME. Functional connectivity of the human brain in Utero. Trends Cogn Sci. 2016;20(12):931–9. 10.1016/j.tics.2016.10.001. 27825537 10.1016/j.tics.2016.10.001 PMC5339022

[117] van Heyningen T, Honikman S, Myer L, Onah MN, Field S, Tomlinson M. Prevalence and predictors of anxiety disorders amongst low-income pregnant women in urban South Africa: a cross-sectional study. Arch Womens Ment Health. 2017;20(6):765–75. 10.1007/s00737-017-0768-z. 28852868 10.1007/s00737-017-0768-z PMC6086488

[118] Verburg C, Griez E, Meijer J. Increase of panic during the second part of pregnancy. Eur Psychiatr. 1994;9(5):260–1. 10.1017/S0924933800003618.

[119] Vereczkey A, Gerencsér B, Czeizel AE, Szabó I. Association of certain chronic maternal diseases with the risk of specific congenital heart defects: a population-based study. Eur J Obstet Gynecol Reprod Biol. 2014;182:1–6. 10.1016/j.ejogrb.2014.08.022. 25216447 10.1016/j.ejogrb.2014.08.022

[120] Vermani M, Marcus M, Katzman MA. Rates of detection of mood and anxiety disorders in primary care: a descriptive, cross-sectional study. Prim Care Companion CNS Disord. 2011. 10.4088/PCC.10m01013. 21977354 10.4088/PCC.10m01013 PMC3184591

[121] Villeponteaux VA, Lydiard RB, Laraia MT, Stuart GW, Ballenger JC. The effects of pregnancy on preexisting panic disorder. J Clin Psychiatry. 1992;53(6):201–3. 1607348

[122] Viswasam K, Eslick GD, Starcevic V. Prevalence, onset and course of anxiety disorders during pregnancy: a systematic review and meta analysis. J Affect Disord. 2019;255:27–40. 10.1016/j.jad.2019.05.016. 31129461 10.1016/j.jad.2019.05.016

[123] Viswasam K, Berle D, Milicevic D, Starcevic V. Prevalence and onset of anxiety and related disorders throughout pregnancy: a prospective study in an Australian sample. Psychiatry Res. 2021;297:113721. 10.1016/j.psychres.2021.113721. 33493733 10.1016/j.psychres.2021.113721

[124] Warren SL, Gunnar MR, Kagan J, Anders TF, Simmens SJ, Rones M, et al. Maternal panic disorder: infant temperament, neurophysiology, and parenting behaviors. J Am Acad Child Adolesc Psychiatry. 2003;42(7):814–25. 10.1097/01.CHI.0000046872.56865.02. 12819441 10.1097/01.CHI.0000046872.56865.02

[125] Warren SL, Racu C, Gregg V, Simmens SJ. Maternal panic disorder: infant prematurity and low birth weight. J Anxiety Disord. 2006;20(3):342–52. 10.1016/j.janxdis.2005.02.007. 16564437 10.1016/j.janxdis.2005.02.007

[126] Weinberg MK, Beeghly M, Olson KL, Tronick E. Effects of maternal depression and panic disorder on mother-infant, interactive behavior in the face-to-face still-face paradigm. Infant Mental Health J. 2008;29(5):472–91. 10.1002/imhj.20193. 10.1002/imhj.20193 PMC3125719 21731149

[127] Wenzel A, Gorman LL, O’Hara MW, Stuart S. The occurrence of panic and obsessive compulsive symptoms in women with postpartum dysphoria: a prospective study. Archives Women’s Mental Health. 2001;4(1):5–12. 10.1007/s007370170002.

[128] Wilson KR, Prior MR. Father involvement and child well-being. J Paediatr Child Health. 2011;47(7):405–7. 10.1111/j.1440-1754.2010.01770.x. 20598076 10.1111/j.1440-1754.2010.01770.x

[129] Wisner KL, Peindl K, Hanusa BH. Relationship of psychiatric illness to childbearing status: a hospital-based epidemiologic study. J Affect Disord. 1993;28(1):39–50. 10.1016/0165-0327(93)90075-u. 8326079 10.1016/0165-0327(93)90075-u

[130] Wisner KL, Peindl KS, Hanusa BH. Effects of childbearing on the natural history of panic disorder with comorbid mood disorder. J Affect Disord. 1996;41(3):173–80. 10.1016/s0165-0327(96)00069-9. 8988449 10.1016/s0165-0327(96)00069-9

[131] Wittchen H-U, Jacobi F. Size and burden of mental disorders in Europe–a critical review and appraisal of 27 studies. Eur Neuropsychopharmacology: J Eur Coll Neuropsychopharmacol. 2005;15(4):357–76. 10.1016/j.euroneuro.2005.04.012. 10.1016/j.euroneuro.2005.04.012 15961293

[132] Woods SM, Melville JL, Guo Y, Fan M-Y, Gavin A. Psychosocial stress during pregnancy. Am J Obstet Gynecol. 2010;202(1):e611–7. 10.1016/j.ajog.2009.07.041. 10.1016/j.ajog.2009.07.041 PMC2811236 19766975

[133] Woolhouse H, Brown S, Krastev A, Perlen S, Gunn J. Seeking help for anxiety and depression after childbirth: results of the maternal health study. Arch Womens Ment Health. 2009;12(2):75–83. 10.1007/s00737-009-0049-6. 19214705 10.1007/s00737-009-0049-6

[134] Yonkers KA, Gilstad-Hayden K, Forray A, Lipkind HS. Association of panic disorder, generalized anxiety disorder, and Benzodiazepine Treatment during pregnancy with risk of adverse birth outcomes. JAMA Psychiatry. 2017;74(11):1145–52. 10.1001/jamapsychiatry.2017.2733. 28903165 10.1001/jamapsychiatry.2017.2733 PMC5710298

