# Supplementary material for: Panic disorder during pregnancy and the first three years after delivery: a systematic review
Source: BMC Pregnancy Childbirth. 2025 Jan 17;25:36. doi: 10.1186/s12884-024-07127-1 (PMC11740555; doi:10.1186/s12884-024-07127-1)
Supplement: Supplementary file 2 — Supplementary Material 2. [file 12884_2024_7127_MOESM2_ESM.docx]

Supplemental Table 1: Prevalence of Panic Disorder in the Peripartum Period

| Adewuya et al. (2006)  *Nigeria* | 344 (172 pregnant women, 172 non-pregnant controls) | case control | MINI (DSM-IV)  t test, Χ^2^ test | Pregnant women: third trimester | - PD point prevalence did not differ significantly between pregnant women (5.2%) and controls (1.7%).  - The rate of any anxiety disorder in pregnant women was elevated (39%) compared with the non-pregnant population (16.3%). |
| --- | --- | --- | --- | --- | --- |
| Farias et al. (2013)  *Brazil* | 239 | cross sectional (convenience sample) | MINI (DSM-IV)  t test, U test, adjusted prevalence ratios | first trimester | - PD point prevalence 0.4%. |
| Giardinelli et al. (2012)  *Italy* | 760 | prospective | SCID (DSM-IV), STAI, EPDS  U test, Χ^2^ test, Pearson correlation for risk factor correlation, odds ratios for specific risk factors | baseline third trimester, follow-up screening 12 weeks postpartum | - PD prevalence 5.4%.  - Antenatal depressive and anxiety symptoms appeared to be as common as postnatal symptoms. |
| Guler et al. (2008b)  *Turkey* | 512 | cross sectional (convenience sample) | SCID (DSM-IV)  Kruskal-Wallis test, U test, Χ^2^ test | third trimester | - PD point prevalence 2.5%. |
| Marchesi et al. (2013a),  Marchesi et al. (2013b)  *Italy* | 324 | prospective | DSM-IV: PRIME-MD and HADS  logistic regression | first trimester: baseline, on average 5 follow-ups in pregnancy | - PD prevalence throughout pregnancy 7.5%, with more than half (57.1%) showing depressive comorbidity.  - Alexithymia did not represent a personality trait that increases the risk of developing PD in pregnancy, but was a state dependent phenomenon. |
| Smith et al. (2004)  *USA* | 387 | cross sectional (convenience sample) | MINI (DSM-IV), PHQ, medical records  Fisher’s exact test, Χ^2^ test, logistic regression | any point in pregnancy (first contact with OB/GYN services) | - PD point prevalence 2%.  - All screened PD patients were already in specific panic treatment (pharmaco- and/or psychotherapeutic). |
| Uguz et al. (2010)  *Turkey* | 416 (309 pregnant women, 107 non-pregnant controls) | case control (convenience sample) | SCID (DSM-IV)  Fisher’s exact test, U test, Χ^2^ test | any point in pregnancy (on average 23^rd^ gestational week), at inclusion for controls | - No difference in PD prevalence (1.9%) in pregnant women vs. controls (0.9%). |
| Wenzel et al. (2005)  *USA* | 146 | cross sectional (convenience sample) | SCID (DSM-III), BAI, BDI-II, PSWQ, SIAS, DAS  linear and logistic regression | 6-8 weeks postpartum | - PD prevalence 1.4%, with 50% onset postpartum. No additional subsyndromal cases. |
| Zar et al. (2002)  *Sweden* | 386 | cross sectional (at-risk sample: antenatal women screened positively for anxiety symptoms) | screening: W-DEQ, customized anxiety questionnaire, diagnosis: ADIS-R (DSM-IV)  t test, U test, Χ^2^ test, ANOVA | gestational week 32 | - PD point prevalence 1.3%.  - There was no association of PD with tokophobia. |
| ANOVA: Analysis of Variance; ADIS-R: Anxiety Disorders Interview Schedule – Revised; BAI: Beck Anxiety Inventory; BDI-II: Beck Depression Inventory V.2; DAS: Dyadic Adjustment Scale; DSM: Diagnostic and Statistical Manual of Mental Disorders; EPDS: Edinburgh Postnatal Depression Scale; HADS: Hospital Anxiety and Depression Scale; MINI: Mini-International Neuropsychiatric Interview; PHQ: Patient Health Questionnaire; PRIME-MD: Primary Care Evaluation of Mental Disorders; PD: Panic Disorder; PSWQ: Penn State Worry Questionnaire; SCID: Structured Clinical Interview for the DSM; SIAS: Social Interaction Anxiety Scale; STAI: State-Trait Anxiety Inventory; t test: Student’s t test for continuous variables; U test: Mann-Whitney U test for non-parametric variables; Χ^2^ test: Chi Square test for categorical variables; W-DEQ: Wijmer Delivery Expectancy/Experience Questionnaire. | | | | | |
| Comprehensively summarized in two systematic reviews/meta-analyses (Goodman et al. 2016; Viswasam et al. 2019) and thus excluded from our review of peripartum PD. | | | | | |
